# A Review of Microscale, Rheological, Mechanical, Thermoelectrical and Piezoresistive Properties of Graphene Based Cement Composite

**DOI:** 10.3390/nano10102076

**Published:** 2020-10-21

**Authors:** Sardar Kashif Ur Rehman, Sabina Kumarova, Shazim Ali Memon, Muhammad Faisal Javed, Mohammed Jameel

**Affiliations:** 1 Department of Civil Engineering, Abbottabad Campus, COMSATS University Islamabad, Abbottabad 22060, Pakistan; arbabfaisal@cuiatd.edu.pk; 2 Department of Civil and Environmental Engineering, School of Engineering and Digital Sciences, Nazarbayev University, Nur-Sultan 010000, Kazakhstan; Sabina.kumarova@nu.edu.kz; 3 Department of Civil Engineering, King Khalid University, Abha 61421, Saudi Arabia; Jamoali@kku.edu.sa

**Keywords:** graphene, cement composite, characterization, rheological, application, energy harvesting

## Abstract

Extensive research on functionalized graphene, graphene oxide, and carbon nanotube based cement composites has been carried out to strengthen and overcome the shortcomings of construction materials. However, less literature is available on the pure graphene based cement composite. In this review paper, an in-depth study on a graphene-based cement composite was performed. Various structural forms of graphene and classifications of graphene-based nanomaterial have been presented. The dispersion mechanism and techniques, which are important for effective utilization in the construction industry, are reviewed critically. Micro-scale characterization of carbon-based cement composite using thermogravimetric analysis (TGA), infrared (IR) spectroscopic analysis, x-ray diffractometric (XRD) analysis, and morphological analysis has also been reviewed. As per the authors’ knowledge, for the first time, a review of flow, energy harvesting, thermoelectrical, and self-sensing properties of graphene and its derivatives as the bases of cement composite are presented. The self-sensing properties of the composite material are reported by exploring physical applications by reinforcing graphene nanoplatelets (GNPs) into concrete beams.

## 1. Introduction

The construction industry makes a significant contribution to economic growth in every part of the world. Every year, 20–35 billion tons of concrete is used globally, making it the most widely used construction material [1], and its advantages, including high strength, durability, fire resistance etc., increase the consumption of concrete [2,3]. However, the main drawbacks of concrete are known to be its brittleness and low tensile strength [4]. In order to overcome the shortcomings of the concrete, researchers used different materials and techniques [5,6,7,8,9,10]. Chemical admixtures [5,6,7,8], supplementary cementitious materials [11,12,13,14], and fibres [15,16,17,18,19] were used to halt the propagation of micro-cracks and improve tensile strength. The size of these fillers ranged from the macro-scale to micro and nano scales. Currently, advancements in nanotechnology made it possible to control nano size cracks (pores with diameter <20 nm) before micro size cracks are developed [20]. Hence, nanomaterials like graphene, carbon nanotubes, and graphene oxide were studied by many researchers [21,22,23].

The literature on the review of nanotechnology in concrete [24] has highlighted several key findings. As for nano-reinforcements, the addition of carbon nanotubes/nanofibers (CNT/CNF) is widely recognised as an appropriate way to enhance the mechanical properties of the cement composite and to resist crack propagation. Nanotechnology is considered to be effective in terms of compatibility, cost and safety. Fraga et al. [25] observed that the incorporation of finely distributed CNTs in cement matrix reduced crack development. In addition, a small amount of multiple walls concentrically arranged in CNTs are able to enhance mechanical properties significantly in terms of tensile strength, brittleness and strain capacity. Han et al. [26] reported the enhancement mechanism of CNTs/CNFs and graphene nanoplatelets (GNPs). CNTs/CNFs have remarkable effect on the mechanical characteristics of cement-based composite, such as pore filling between hydration products (ettringite and CSH gel) which as a result enhances bond strength between the matrix and CNTs/CNFs while the GNPs showed an augment of the compactness and the homogeneity of hardened cementitious composite. Also, the addition of CNTs/CNFs and GNPs led to the improvement of piezoelectric and dielectric properties.

Qureshi and Panesar [27] in their literature review emphasized a nanofibrous material, graphene oxide (GO), which potentially improves the behavior of cement-based materials. According to the authors [27], addition of 0.05% of GO by weight of cement in portland cement paste increased its compressive strength and flexural strength by 15–33% and 41–59%, respectively. Moreover, a 70.5% increase in flexural strength was found when GO was dispersed with a superplasticizer. The authors also underlined the need to determine the effect of GO on cement hydration process, life-cycle cost and carbon release. Yang et al. [28] reviewed the size of GO particles and observed that GO of the smaller size showed a better performance when compared to the larger size. They also reported the effect of hybrid GO cement-based composite materials and stated that cement composites with both GO and CNTs perform better than a cement composite with only GO or CNTs. This was due to the reason that CNTs in GO solution are dispersed better because of the huge electrostatic repulsion in the CNTs/GO mix. The authors also considered it obligatory to concentrate on the synergic effects of nanomaterials and GO, since dispersion issue restricts the application of nanomaterials in civil engineering. The application of graphene and GO in geopolymer cement composites has been reviewed by [29]. The authors concluded that the experimental results of addition of graphene in geopolymers cement composite reported by different researchers vary and are inconsistent. This might be related to different ways of treating graphene nanomaterials which will results in more interference factors in addition to varying content of aluminium-silicon of geopolymers. Recently, Zhao et al. [30] reviewed the impact of GO on cement composite. The authors observed inconsistency amongst the various published literature due to complex nature of hydrated cement matrix and varying characteristics of graphene oxide.

In this review article, a detailed study on the graphene-based cement composite is performed. A brief explanation of graphene nanostructures is presented in Section 2. In Section 3, various structural forms of graphene are discussed, and classification of graphene-based nanomaterial is presented. As the dispersion of graphene-based nanomaterials is one of the substantial challenges for their employment in the construction industry, dispersion techniques along with mechanisms are reviewed in Section 4. Micro-scale characteristics of the graphene and its derivatives-based cement composite is essential as it helps in scientific understanding of the material. Hence, in Section 5, various characterization techniques, including thermogravimetric analysis (TGA), infrared (IR) spectroscopic analysis, X-ray diffractometric (XRD) analysis, and morphological analysis, are reviewed. In Section 6, Section 8 and Section 9, for the first time per the authors’ best knowledge, a review of flow properties, energy harvesting, thermoelectrical, and self-sensing properties of graphene based cement composites are presented. Finally, the research gaps are highlighted in Section 10 while conclusions are presented in Section 11.

## 2. Brief Description of Graphene Nanostructures

**Figure 1:**
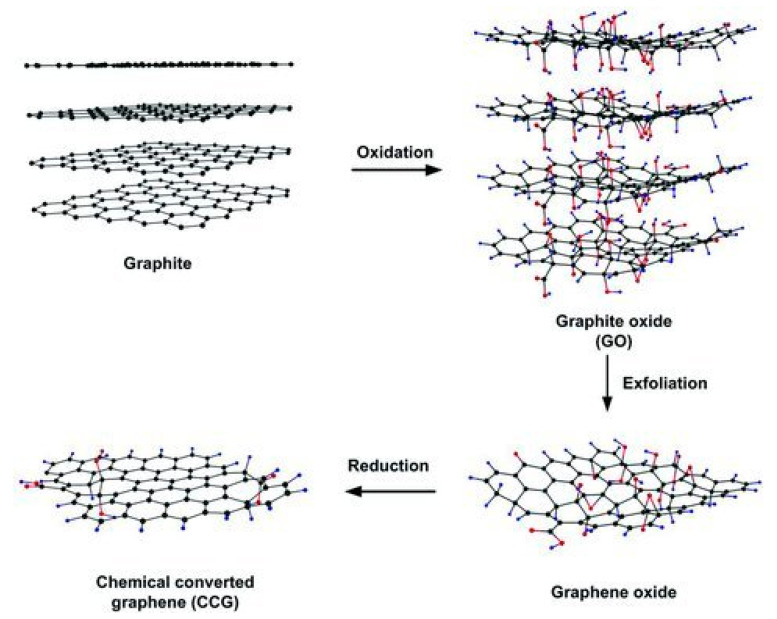
Preparation of graphene nanosheets from graphite. Reproduced with permission from [37], John Wiley and Sons, 2011.

Graphene is a single layer carbon sheet and one of the most promising nanofiller used to enhance cementitious materials [31,32]. Practically, multi-layered GNPs are very commonly used since they can be easily manufactured from graphite oxide or graphite. GNPs consist of layers of graphene having a thickness from 3 to 100 nm. Thus, their morphological structure makes it a remarkable reinforcing material [33]. GO is a layered material, which is oxidized from graphite, and oxygen particles are interspersed on the edges and its basal surfaces. In fact, graphene sheets are naturally available and it is only required to exfoliate them from the graphite [34]. The exfoliation of graphite into GNPs can be made by using chemical and mechanical techniques [35]. The mass scale production of graphene is possible by means of chemical oxidation and reduction of graphite [36]. This method is considered to be faster, easier, more scalable, economic, facile, and dynamic as compared with other methods [36]. Figure 1 presents the chemical procedure for production of graphene from naturally available graphite.

This section may be divided by subheadings. It should provide a concise and precise description of the experimental results, their interpretation as well as the experimental conclusions that can be drawn.

## 3. Classification of Graphene Based on Nanostructure

**Figure 2:**
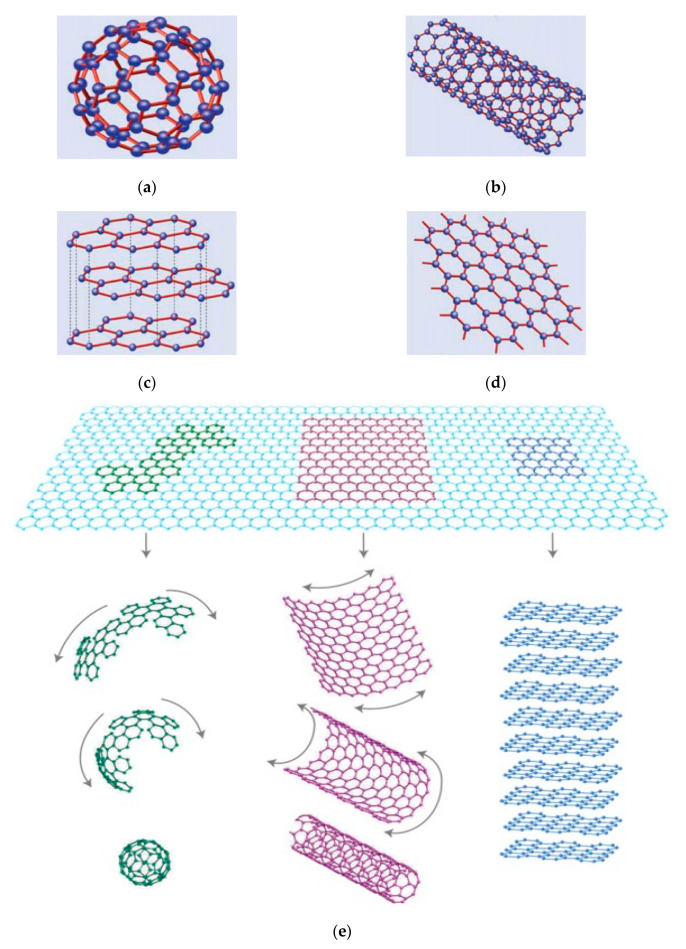
Various structural form of graphene; (**a**) wrapped honeycomb structure of zero-dimensional graphene nanoparticle; (**b**) Rolled honeycomb structure forms of one-dimensional graphene; (**c**) single planar two-dimensional graphene sheet; (**d**) stacked honeycomb structure of three-dimensional graphite, and (**e**) extraction of various structural forms from 2D graphene sheet. Modified from [39,40], Elsevier, 2010.

These newly developed engineered nanomaterials are characterised by their morphology: zero-dimensional (0D) nanoparticles (spherical shape and low aspect ratio), i.e., carbon black; one-dimensional (1D) fibers (straight and high aspect ratio), i.e., carbon nanotubes; and two-dimensional (2D) sheets, i.e., graphene and GO [21]. Figure 2 shows the schematic of these nanomaterials. These engineered materials are used in the construction industry to overcome weaknesses of building materials, i.e., cement paste, mortar and concrete. Besides enhancing the mechanical abilities of cement composites, the addition of the nanomaterials also improves their electrical, thermal, and electromagnetic properties [38].

### 3.1. Zero Dimensional Graphene Nanoparticles

Molecules, which consist of wrapped graphene by means of the introduction of pentagons on the hexagonal lattice are called zero dimensional nanoparticles. These allotropes of carbon were discovered by Kroto et al. [41]. They are like Buckyball’s; common examples are Fullerenes (C60) and Carbon Black (CB). Several researchers have explored the mechanical and electrical properties of carbon black cement composite as well as its applications for structural health monitoring. According to Xi et al. [42], CB particles having about 33 nm diameter provides cheaper solution for piezo-resistive effects as compared with carbon fibers mixed concrete. They found that CB filled cement matrix is promising candidate for strain sensing. Gong et al. [43] observed that piezoelectric sensitivity of the cement composite enhanced dramatically by addition of 1% volume of CB. Wang et al. [44] dispersed the CB particles in high density polyethylene matrix and silicone rubber, respectively. They also presented a mathematical piezoresistivity model of the CB filled composite material based on the general effective medium theory. The authors found that the results predicted by the mathematical model were in alignment with the experimental results. CB particles are also found to interact with air-entraining admixtures and smaller particle sizes with more surface area have shown optimum interaction results [45]. Figure 2a presents the wrapped honeycomb structure and schematic of zero-dimensional graphene nanoparticle. Zero-dimensional nanoparticles lack the ability to arrest micro-cracks due to non-uniform mixing, a low aspect ratio, and the formation of weak zones in concrete, especially when used in a large amount.

### 3.2. One Dimensional Graphene Nanotubes

Compared to zero-dimensional nanoparticles, spherical shape one-dimensional nanofibers have a high aspect ratio i.e., carbon nanotubes. Exfoliated GNP (xGnP) and carbon nanotubes (CNT) share the same chemical structure [32]. CNTs are carbon allotropes of cylindrical shape, made of rolled graphene layers. Based on the number of walls, CNTs are classified as single wall CNTs (SWCNTs) and multi walls CNTs (MWCNTs), i.e., 10–100 walls. The diameter of SWCNTs varies from 1 nm to 3 nm while the diameter of MWCNTs varies from 5 nm to 50 nm. [46]. MWCNTs have a surface area of around 400 m^2^/g and aspect ratio of more than 1000, due to varying length of carbon nanotubes [26]. CNTs have high elastic modulus of 1TPa, strength of 10–60 GPa for SWCNTs and 50–500 GPa for MWCNTs, and electrical resistance of 5–50 µΩcm. Such impressive properties of CNTs enhanced the properties of cementitious materials, when mixed with cement [47]. Konsta et al. [47] noted 25% rise in the flexural strength of CNTs-cement composite. According to Li et al. [48], when 0.5% functionalized CNTs was added to plain cement concrete, the compressive strength and flexural strength increased by 19% and 25%, respectively as compared with control specimens, while porosity decreased by 64%. Moreover, pores with a size of more than 50 nm in diameter were 82% less as compared with plain cement concrete. Nevertheless, the problem with CNTs is non-uniform dispersion and weak connection between CNTs and the cement matrix. The arrangement of CNTs is complicated because strong Van der Waals forces exist between individual CNTs, which may cause the formation of agglomeration and bundles in the composite. As a result, these agglomerates form defects and limit the influence of CNTs on cement composite [49]. That is the reason why, even after decades of research on CNTs, their full potential as reinforcement has been severely limited [36]. Several researchers [50,51] noted the decline in mechanical properties of the CNT based cement composite because of a non-uniform dispersion, worst workability, higher inhomogeneity, and porosity. Research performed by Cwirzen et al. [50] states that no major effect was recorded on the mechanical properties of the CNT based cement composite mix in contrast with pure cement mix even by using surfactants and achieving uniform dispersion in the mix. This was most probably because of the very low bond strength between CNTs and cement matrix, due to which CNTs were easily pulled out in fractured cement paste specimens.

### 3.3. Two Dimensional Graphene Sheets

In contrast to CNTs, graphene and GO are the two-dimension sheet-like structures and have a considerable surface area. GO has a thickness of a single atomic layer while having lateral thickness reaching to tens of micrometres, which provides large surface area and immense aspect ratio [52]. It has been observed by researchers that by incorporating graphene sheets in cement composite, electrical, mechanical and thermal properties remarkably enhanced [22,53,54]. Pan et al. [55] reported an increase in tensile strength by 78.6%, flexure strength by 60.7% and compressive strength by 38.9% when 0.03% dosage of GO by weight of cement was incorporated in cement mortar. At the microscopic level, they observed the flower-like crystals, which enhanced toughness. Moreover, Pan et al. [55] found that 41.7% decline in slump size when 0.05% GO by weight of cement was used in the cement paste mix. The possible reason was considered to be the huge surface area of GO, which reduces the accessible moisture content in mix design from wetting the GO sheets. GO is the carbon antecedent combined with carboxyl, epoxy and/or hydroxyl groups [56]. At nanoscale, the spacing between atoms in GO is almost identical to graphene [56]. Extensive research has been conducted on GO cement composite. However, much less focus has been given to graphene cement composite.

In 2004, Novoselov et al. [31] derived single atom thick crystallites of graphene from bulk graphite. They obtained the graphene layer in a repeated pealing Scotch Tape technique process [31]. According to Zhang et al. [57], by using this method, thickness of graphene up to 300 nm can be achieved. Graphene is known to be the thinnest material [58,59]. Boehm et al. [60] concluded that graphene is one of the carbon allotropes with 2D properties. Figure 2c shows that graphene is arranged in a single planar sheet while Figure 2d shows the stacked honeycomb structure forms of three-dimensional graphite. The structural relationship between graphene and various other forms are shown in Figure 2e.

Recently, the 2D flat graphene sheet has gained an enormous attention in science for its promising and outstanding properties: high intrinsic strength (130 GPa) [59], large specific surface area (2630 m^2^ g^−1^) [61], high thermal conductivity (~5000 Wm^−1^K^−1^) [62,63] and firm Young’s module (~1.0 TPa) [64,65,66]. This unique and tremendous behaviour of graphene opened a new window for a wide range of applications. A large exposed surface area of graphene sheets has a strong capability to make a great physical and chemical bond with the cement matrix. Rafiee et al. [67] mentioned that unzipping the MWCNTs into graphene nanoribbons results in a significant improvement. This was due to an extraordinary increase in interfacial area and geometry of graphene sheets as compared with multi-walled carbon nanotubes. Rafiee et al. [67] also found a 30% increment in Young’s modulus and 22% rise in the ultimate tensile strength of graphene composite against the same amount of multi-walled carbon nanotubes composite. Conversely, graphene has a very high cost of manufacturing. Therefore, the application of graphene is restricted and limited in the construction industry due to production in very small quantities [32].

## 4. Dispersion of Graphene Based Nanomaterials

Dispersion is a primary problem related to fabricating cementitious nanocomposite because Van der Waals force forms an agglomeration of the nanoparticles [68]. Dispersion of nanomaterials in aqueous solution is an important step and significantly alters the final results [69,70]. In aqueous solution, nanomaterials tend to precipitate or float on the surface. Naturally, graphene is hydrophobic, and it forms the agglomerates in aqueous solution, causing non-uniform dispersion [71]. According to Hamann and Clemens [72], a weak dispersion will result in the formation of a defect in the composite matrix and restrain the effect of the nanoparticles. Most of the studies about the dispersion of graphene and graphite nanoparticles were directed to surface adjustment, such as oxidation, or the inclusion of other nanoparticles in the suspension [73], oxidation of GNPs to form GO and then reduction to GO particles and finally, dispersed them in water. In addition, oxygen-containing functional groups on the GO could result in stable dispersions due to electrostatic repulsion between the oxidized GO particles [74]. Peyvandi et al. [75] noted that covalent surface modifications cause destructive effects on the atomic structure of the graphite nanoplatelets and are able to reduce the strength of these nanoparticles. In order to preserve the structure of the graphene flakes, other methods of dispersion are required. Still, this area is relatively new and least research on the dispersion of non-covalently modified GNPs in water has been conducted. Hence, to improve the dispersion of graphene flakes keeping the atomic structure safe in the cement composite, numerous techniques and the results of various solvent types have been reviewed in this section.

### 4.1. Dispersion Using Dispersant

**Figure 3:**
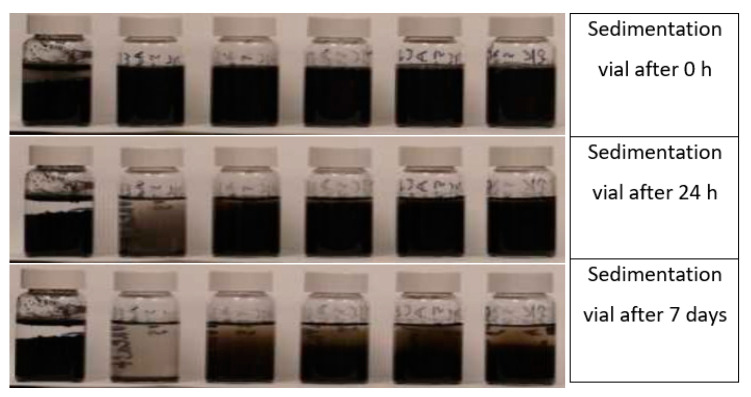
Effect of the presence of WRA to the dispersion of graphene sedimentation at different times; graphene sedimentation jars with WRA-to-GNPs ratios of 0, 1, 1.5, 2, 2.5 and 3 from left to right. Reproduced with permission from [78], Ph.D. Thesis, University of Illinois, 2014.

The following section is regarding the application of dispersant to the Graphene and its derivatives. As graphene flakes are hydrophobic and tend to form coagulation in the aqueous solution, therefore, numerous researchers used different dispersants to obtain a stable suspension. Stankovich et al. [76] used reduced graphite oxide (rGO) flakes with polysodium styrenesulfonate. These graphite nanoparticles, which were coated with the polysodium styrenesulfonate, remained in suspension. Jue et al. [77] noted that using of polyelectrolytes with GNPs in water retains the structure of the GNPs and provides a complete utilization of the features of these nanoparticles. Wotring [78] evaluated the performance of GNPs dispersion in water with high-range water-reducing admixture (WRA). Water-cement (w/c) ratio remained as 0.5, graphene dosage was 0.1% by weight of cement paste and AdvaCast 575 polycarboxylates based high range WRA in the range of 0–10 times the weight of graphene flakes were used. According to Wotring [78], the stability of graphene in the solvent was preserved by the WRA. When the dosage of water reducing agents increased, the stability also increased as shown in Figure 3. By visual observations, it was found that the WRA to GNPs ratio of 3% was stable until seven days, as shown in Figure 3 [78]. Meanwhile, good stability was observed after 24 h for other ratios of WRA to GNPs.

**Figure 4:**
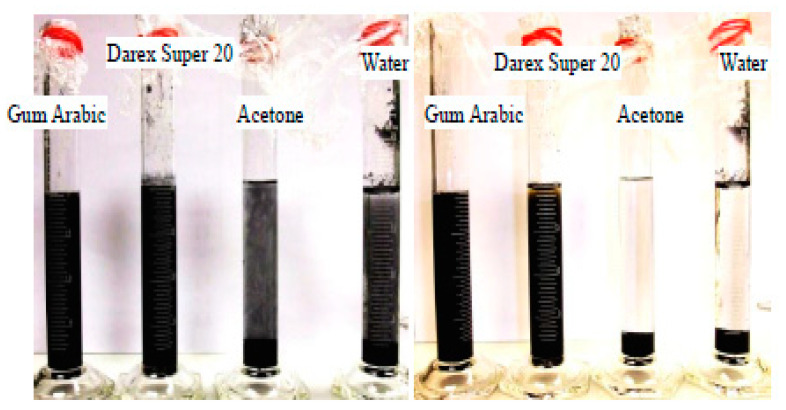
Stability test for graphite suspension in various dispersant of liquid at 5 min (left) and 30 min (right). Reproduced with permission from [79], ME. Thesis, National University of Singapore, 2012.

Sixuan [79] used acetone, particularly Gum Arabic and Darex Super 20 solvent, for the dispersion of graphite nanoplatelets in water solution. The stability and homogeneity of graphite flakes suspensions with respect to time using various dispersing agents were tested by visual inspection. Figure 4 presents the dispersion of graphite flakes in different dispersants. The stability of graphite flakes was assessed by observing the colour of the solution. Usually, the dispersant and distilled water are colourless and after incorporating the graphite, the colour changed to black. Based on Figure 4, the graphite nanoplatelets in Darex Super 20 and Gum Arabic presented good stability and the colour of the solution remained unchanged after 30 min of mixing. However, in acetone and water solution the graphite accumulated at the bottom of the test tube. Gum Arabic is a natural polysaccharide available as white powder and is remarkably soluble in the water. The weak acidity of Gum Arabic makes it reactive in the alkaline cementitious environment, thus leading to a production of surplus water while mixing. Finally, a watery mixture containing light graphite flakes on top was formed. Likewise, Darex Super 20, which is the naphthalene sulfonate-based high water-reducing superplasticizer, exhibited a good stability as dispersant with minimal alteration on the fresh mixture. Therefore, based on test results, Sixuan [79] concluded that Darex Super 20 was found to be the best dispersant among acetone, tap water, and Gum Arabic for the graphite nanoplatelets in the cement matrix.

**Figure 5:**
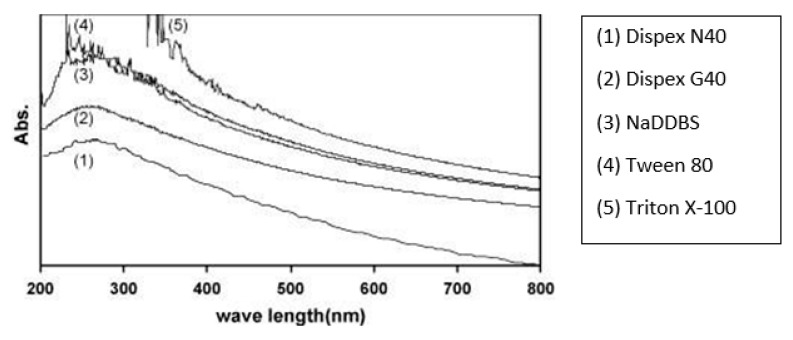
UV–vis absorption spectra of carbon black in presence of various dispersant. Reproduced with permission from [80], Elsevier, 2009.

Sharif et al. [80] examined the dispersion of CB, i.e., zero dimensional graphene in the presence of five dispersants, including Dispex G40, sodium dodecylbenzene sulfonate (NaDDBS, MW = 348.47), Tween 80, *t*-octylphenol decaethylene glycol ether (Triton X-100, MW = 647), and Dispex N40. They concluded that most favourable dispersant was Triton X-100, a surfactant molecule, as it showed the highest absorption peak in the UV-vis absorption spectra, as presented in Figure 5. The affixing of surface-active agents to the surface of carbon nanomaterial is mainly attributed to hydrophobic synergy.

Recently, Silva et al. [81] used isopropanol alcohol with the expanded graphene structures in a ratio of 1:1. The authors found that the combination of multilayer graphene sheets and isopropanol produced excellent dispersion. Han et al. [82] used the polycarboxylate superplasticizer (Sike ViscoCrete 3301E) to disperse multi-layered graphene in aqueous solution and found that the graphene flakes did not form agglomerations.

**Figure 6:**
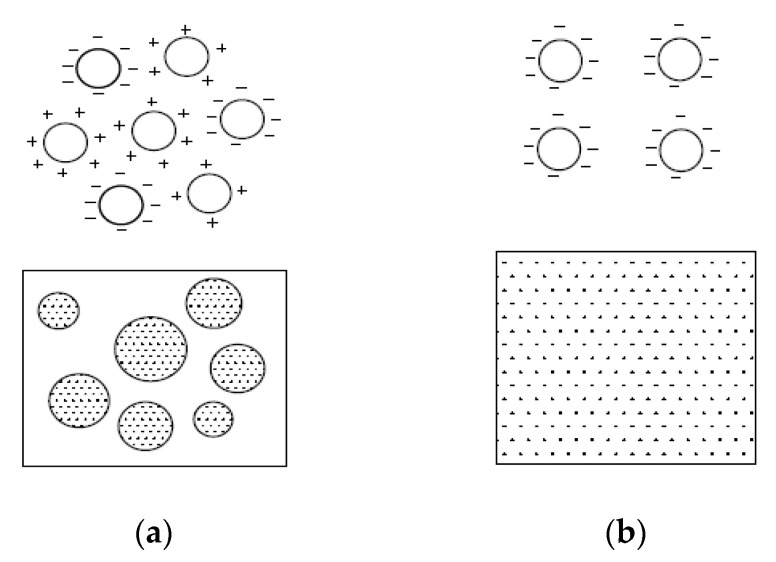
Action of dispersion for graphite nanoparticles; (**a**) schematic diagram of flocculated graphite flakes in aqueous solution; and (**b**) and schematic diagram of uniformly dispersed graphite nanoparticles. Reproduced with permission from [79], ME. Thesis, National University of Singapore, 2012.

The dispersion mechanism for dispersant and graphite nanoplatelets was explained by Sixuan [79]. The authors stated that the organic molecules in the dispersant are negatively-charged and absorbed mainly at the interface of water and graphite. The graphite surface initially possessed the residual charges on their surfaces. When these graphite nanoparticles were mixed in liquid solution, they formed the flocculated structures. The flocculation of the graphite particles occurred due to the electrostatics interactions exerted by the adjacent graphite particles of the opposite charges, as seen in Figure 6a. After that, dispersant was used to neutralize these residual charges and made the entire surface to carry the same charges. Lastly, the particles of graphite nanoplatelets remained fully dispersed in the suspension of the liquid because of the repulsion of the graphite nanoparticles (Figure 6b) [79].

### 4.2. Dispersion Using Ultrasonication

**Figure 7:**
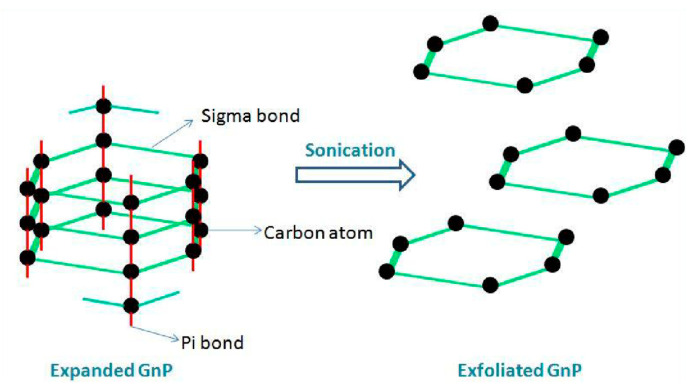
Mechanism of graphene dispersant after ultrasonication from expanded GNP to exfoliated GNP. Reproduced with permission from [79], ME. Thesis, National University of Singapore, 2012.

Another way to deal with dispersion problem with graphene and its derivatives to apply sonication. The sonication is the act of agitation of particles by means of applying energy. The ultrasonication is referred to as the waves having a frequency of more than 20 KHz. The ultrasonic electric generator takes the biggest part of sonication device and generates a signal, normally about 20 KHz, which charges a transducer and it transforms the electric signal to mechanical vibrations. These vibrations are further augmented by the sonicator and transmitted to the probe, which transfers the vibrations to the solution. The quick movement of the probe produces a cavitation event. It takes place when the vibrations generate multiple microscopic bubbles in the solution, some wedged intermolecular space is developed and breaks down continuously under the influence of the weight of the solution. Constant generation and breakdown of thousands of these bubbles develop the robust waves of vibration, which pass through the solution and crush the particles [83]. The energy, which had been transmitted to the GNPs resulted in the collapse of the interlayer π-bond. Hence, exfoliated GNPs can be attained with higher aspect ratio, decreased thickness and improved mobility of particles as shown in Figure 7. The maximum size of the bubble being produced in the liquid is dependent on the frequency of ultrasonication. A low-frequency ultrasonication will generate large size bubbles and vice versa. Higher energy forces are being produced upon the collapse of the large-sized bubbles in the solution [79].

Mehrali et al. [84] applied sonication to GNPs in distilled water with a high-powered probe sonicator. The stability of the GNPs was reported to remain in suspension for 600 h. Han et al. [82] used the ultrasonication for 1 h to achieve the uniform dispersion of cementitious materials with multi-layered graphene (MLGs). Silva et al. [81] employed the isopropanol alcohol blended with the expanded graphite structures in 1:1. The solution was then ultra-sonicated for the next 2 h and achieved excellent dispersion.

### 4.3. Assessment of Dispersion Efficiency Using UV-Vis Spectrometry

**Figure 8:**
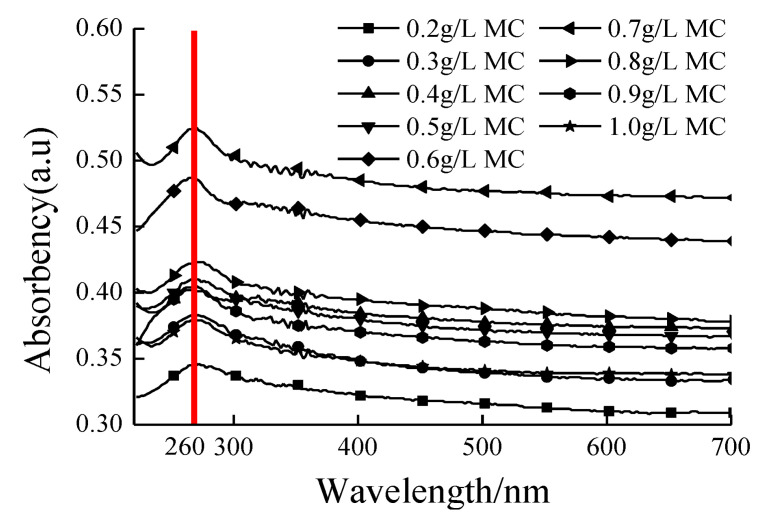
UV-Vis spectroscopy absorbance range of GNPs suspension with different MC amounts. Reproduced with permission from [33], MDPI, 2016.

**Figure 9:**
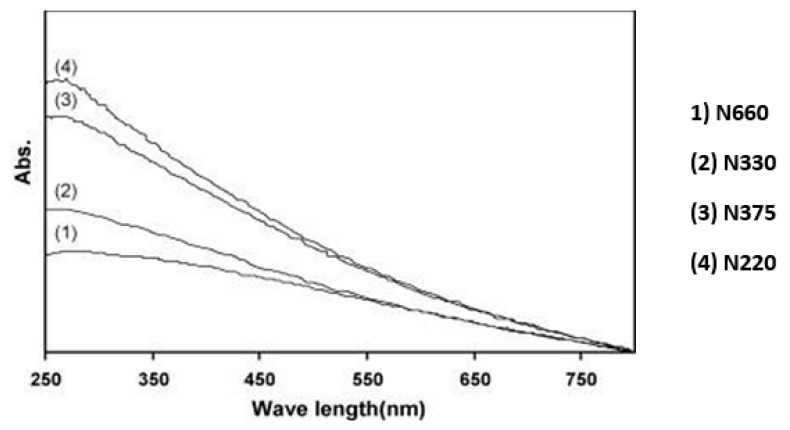
UV–vis absorption spectra for dispersion efficiency of various types of CB in aqueous solution. Reproduced with permission from [80], Elsevier, 2009.

Ultraviolet-visible spectroscopy (UV-vis) is defined as an absorption or reflectance spectroscopy in the UV spectral region. It is also known as a competent method to examine the dispersion of graphene structure, particularly, the GNPs and CNTs. Jiang et al. [85] used the UV-vis measurements for the quantitative evaluation of the colloidal stability of CNTs dispersion. In UV-vis spectral range, carbon nanomaterials showed the absorption characteristics and it is attributed to the electronic changes between the bonding and antibonding π orbital [86]. Jager et al. [87] stated that the σ–σ* transitions are anticipated in the 60–100 nm ultraviolet range, meanwhile the π–π* transitions are observed in 180–280 nm spectrum. Due to this reason, this method has been utilized by the many researchers to evaluate the dispersion of rGO as shown in Table 1. Wang et al. [33] used graphene flakes in cement composite with w/c of 0.35 and 0.05% GNPs by weight of cement with different concentrations of dispersant Methylcellulose (MC) within the range of 0.2–1.0 g/L. It was noted (Figure 8) that, for different mixes, the highest peak of GNPs-suspension was found at a wavelength of 260 nm in UV-vis spectra. Similarly, a peak of the absorption at 270 nm was observed in the UV-vis absorption spectrum of graphene, which is generally regarded as the agitation of the π-plasmon of the graphitic structure [88]. Moreover, Aunkor et al. [88] research state that the absorption peak value is a function of the concentration of dispersed graphene sheets. Sharif et al. [80] investigated the dispersion of CB applying UV-vis spectroscopy and determined the rise in UV-vis absorption related to the surface area of nanomaterials (Figure 9). They used four types of carbon black with varying surface areas and determined the highest dispersion values for CB having the highest surface area.

Therefore, it can be concluded from the literature of the dispersion section that the ultra-sonication technique provides uniform dispersion to graphene flakes. The use of dispersant, i.e., polycarboxylate based high range water reducing admixture will help in exfoliation of graphene flakes. Additionally, the UV-vis spectroscopy method is extensively applied to monitor and assess the dispersion of nanomaterials in aqueous solution.

## 5. Characterization of Graphene Cement Composite

Characterization refers to the procedures by which the material’s properties and structure are explored and measured. The graphene-based nanomaterials interact with hydrated cement products and influence the hardened properties of the composite material. Thus, it is important to study the micro-scale characterization of graphene-based cement composite. In this section, the characteristics of graphene-based cement composite are explored by (a) thermogravimetric analysis (TGA), (b) infrared spectroscopic analysis, (c) X-ray diffractometric (XRD) analysis, and (d) morphological analysis.

### 5.1. Thermogravimetric Analysis (TGA)

**Figure 10:**
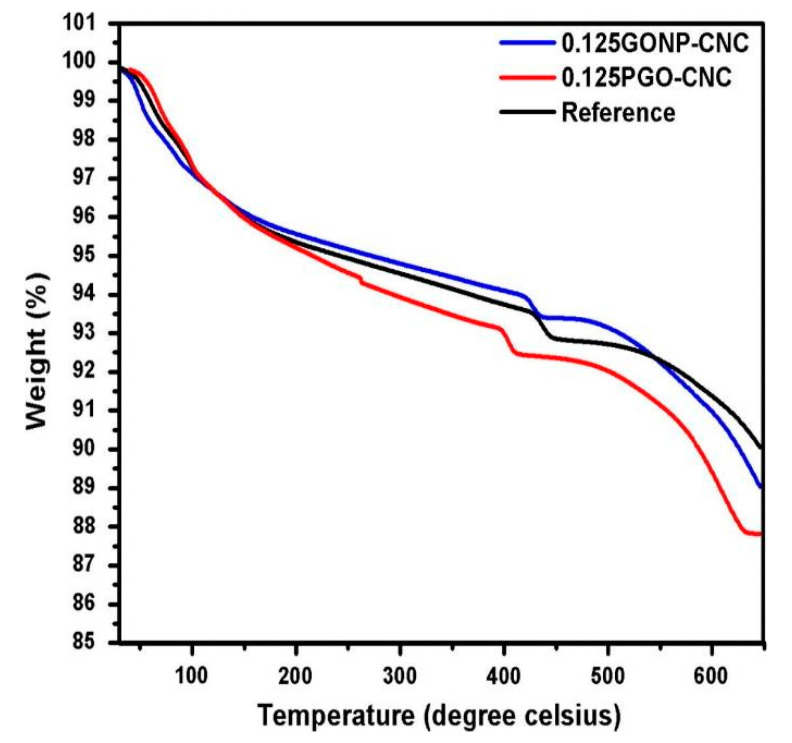
TGA curves of plain mix, pristine graphene oxide mortar composite and graphene oxide mortar composite after 90 days of curing. Reproduced with permission from [107], Elsevier, 2016.

Thermal analysis is a method which estimates the change in the materials properties depending on the temperature [106]. The effects of pristine graphene oxide (PGO) and graphene oxide nanoplatelets (GONPs) (produced from ball-milling) were investigated by Sharma and Kothiyal [107]. They used 0.10% and 0.125% of PGO and GONPs by weight of cement in mix design with a w/c ratio of 0.45. Figure 10 presents the TGA curves for the control sample, 0.125% of PGO-cement mortar nanocomposites (PGO-CNCs) and GONPs-CNCs obtained after 90 days of curing. The weight loss corresponding to CH in the control mix, pristine graphene oxide (0.125PGO-CNC) and GO nanoplatelets (0.125GONP-CNC), appeared to be 12.7%, 10.8%, and 5.3% respectively. The final weight loss in the TGA curve of GO-based cement mortar was slightly greater as compared with the control mix due to the inherent thermal conductive properties of GO as shown in Figure 10 [107].

**Figure 11:**
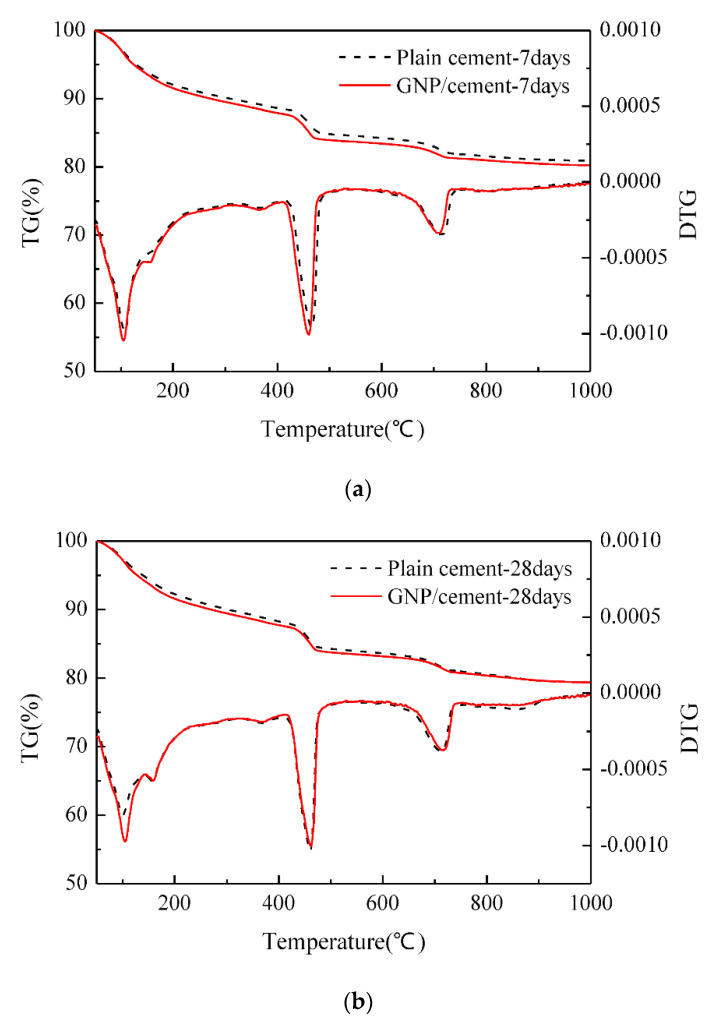
TGA curves of cement composites with and without GNPs (**a**) 7 days, and (**b**) 28 days. Reproduced with permission from [33], MDPI, 2016.

Wang et al. [33] observed the variation in the TGA curve after the addition of graphene nanocomposites to the cement paste. Figure 11 showed the TGA curve for cement composites with and without GNPs at 7-day and 28-day respectively. Both samples showed similar trends at seven and 28 days, however, the graphene flakes accelerated the hydration process of cement. On the 7th day, the amount of amorphous phases, i.e., CSH and calcium hydroxide in GNP-cement composite was greater than the control cement phase. However, the content of CSH gel and calcium hydroxide in both samples was nearly equal after 28 days of curing. It was concluded that GNPs enhanced the hydrated cement products at an early age.

### 5.2. Infrared Spectroscopic Analysis

**Figure 12:**
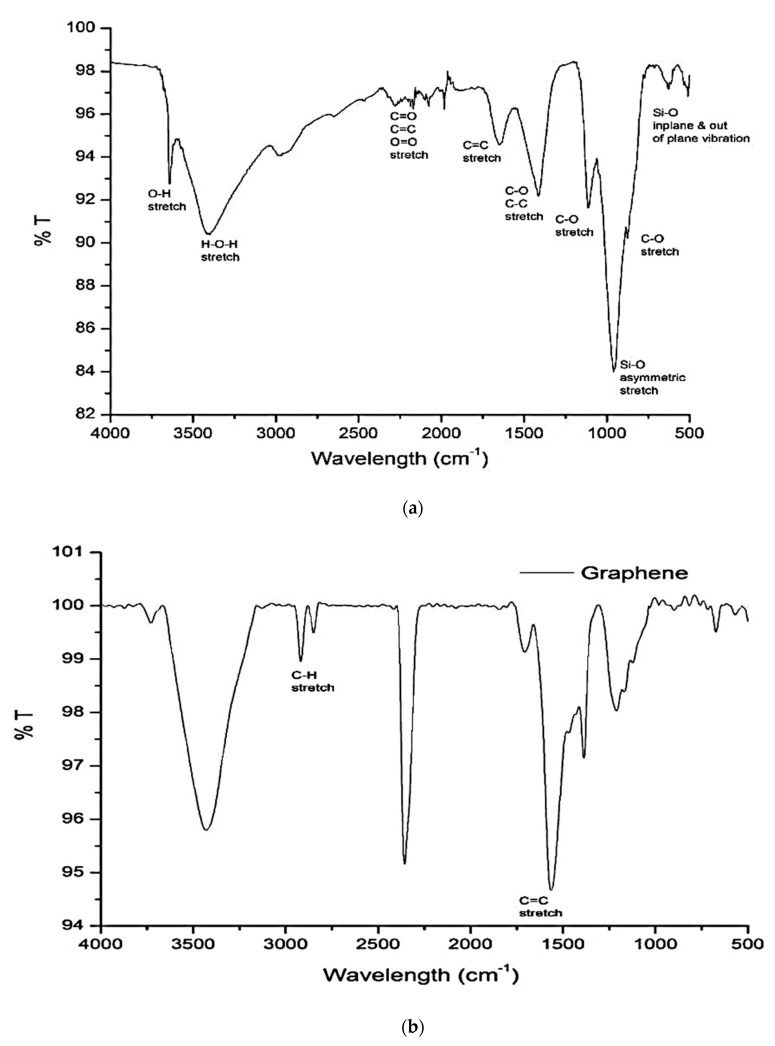
Analysis of FTIR spectra of (**a**) control sample adapted from [48] Elsevier, 2005 and (**b**) graphene adapted from [88], RSC Advances, 2015.

Vibrational spectroscopy is an approach for the assessment of the molecular structure. It provides useful information about possible chemical and physical interaction [108]. FTIR spectra of 28-day cement paste and graphene are given in Figure 12. The spectrum of control sample is shown in Figure 12a while the observed maximum peak values are listed in Table 2 [108,109,110,111]. In graphene spectra (Figure 12b), besides these peaks, two additional peaks are observed at 1570 cm^−1^ (sp2 hybridized C=C), 2918 cm^−1^ and 2850 cm^−1^ (symmetric and asymmetric stretching vibration of –CH_2_), pointing to the sp2 network [112]. According to Mollah et al. [108], FTIR spectra can be divided into three regions; (a) water region (>1600 cm^−1^); (b) the sulphate region (1100–1150 cm^−1^); and (c) the material region (<1000 cm^−1^). Spectral data vary by the incorporation of different nanomaterials and assessments of these transitions will yield useful information. Shifting of these bands implies the stronger bonding, formation of hydrated products and polymerization in hydrated products [108,113] while the variation in the absorbance intensities provides information about the quantity of material [113].

**Figure 13:**
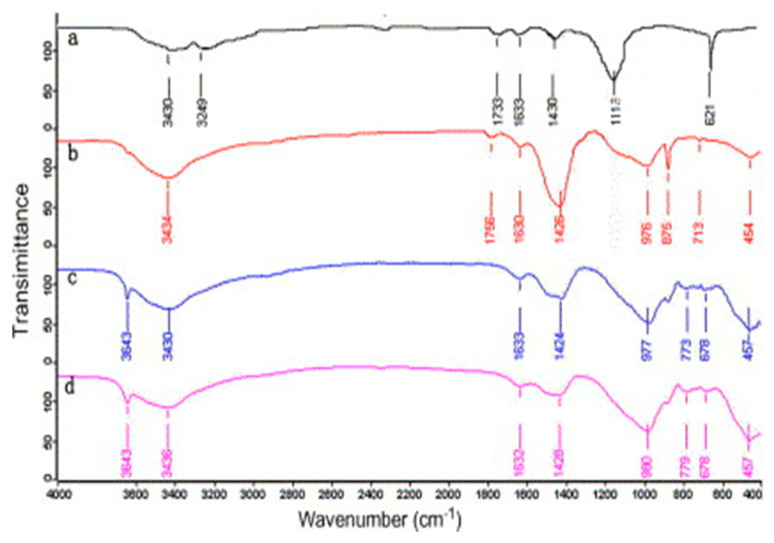
FT-IR spectra of various cement composites at age of 90 days. (**a**) carboxylic carbon nanotubes; (**b**) carboxylic carbon nanotubes cement paste; (**c**) carbon fibres cement paste and; (**d**) plain cement paste. Reproduced with permission from [48], Elsevier, 2005.

Geng et al. [48] employed FTIR analysis on three different blends of cement paste. The w/c was maintained at 0.45 and CNTs and carbon fibers were added 2% by weight of cement. Figure 13 shows four FTIR spectra, and three spectra are associated with cement pastes prepared with and without CNTs and carbon fibers while the remaining one is related to treated carbon nanotubes. The authors noted that untreated carbon fibers cement paste spectra were similar to plain cement paste as presented in Figure 13c. Hence, no chemical interaction and new phase formation were recorded. The spectra in Figure 13a displayed the peaks at 1733 cm^−1^ and 1118 cm^−1^ corresponds to C=O stretching of carboxylic acid and C-OH stretch of hydroxyl. These peaks confirmed that various oxygen-containing groups are attached to the surface of CNTs. When these surface treated CNTs were added to cement paste, a chemical interaction took place, as shown in Figure 13b. A positive shift in a spectral peak at 1756 cm^−1^ by 22 cm^−1^ indicated the probable existence of carboxylate while the disappearance of peak at 3643 cm^−1^ specified the chemical synergy between hydrated cement products with oxygen groups of carboxylic acid attached with the CNTS. In addition, the variation in spectral shape in the CSH region indicates the variation in CSH phases due to the functionalization of carbon nanotubes.

**Figure 14:**
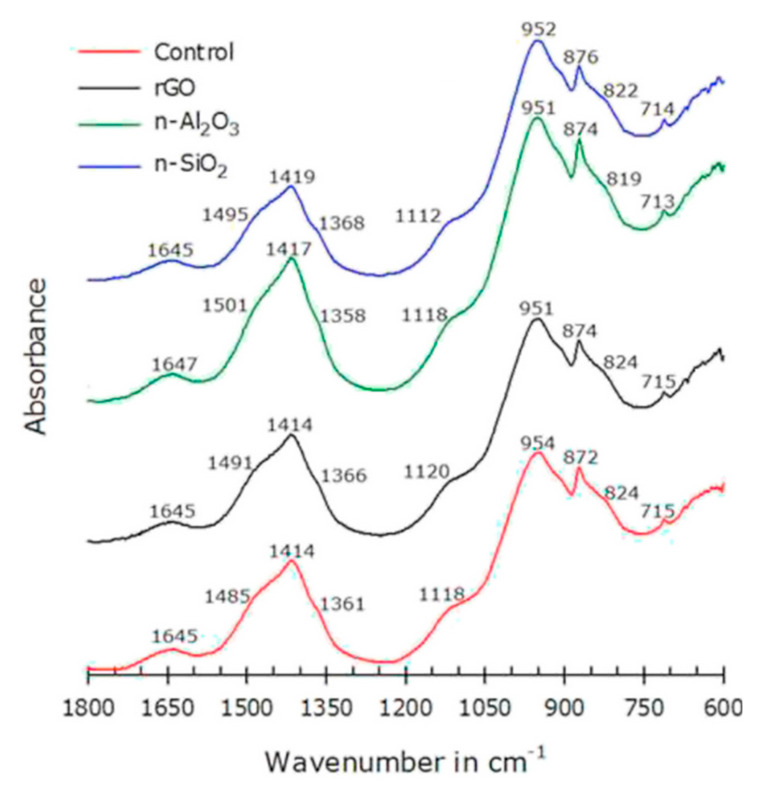
FTIR curve of the various pastes cured for 28 days. Reproduced with permission from [114], Elsevier, 2016.

The effect of rGO, n-Al_2_O_3,_ and n-SiO_2_ to cement composite was investigated by Murugan et al. [114]. Figure 14 presents the FTIR spectra of these cement pastes. The authors did not notice any major difference in FTIR spectra. In these spectra of cement pastes observed after 28 days of curing, free water peak found at ~1645 cm^−1^ was attributed to O–H bend, ettringite peak at ~1118 cm^−1^ was due to S–O stretch, silicates peaks at ~950 cm^−1^ was due to Si–O asymmetric stretch. Calcite peaks, which are indicative of carbonation, were recorded in all mixes at ~1414 cm^−1^ and ~874 cm^−1^, attributed to C–O stretching and C–O bend vibration respectively.

### 5.3. XRD Analysis

**Figure 15:**
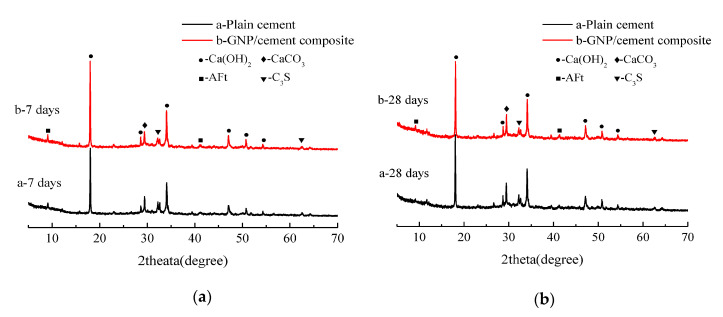
XRD patterns of GNP cement composite and plain cement at (**a**) 7-day and (**b**) 28 days. Reproduced with permission from [33], MDPI, 2016.

X-ray diffractometer was broadly adapted for determining the crystalline structure of materials [106]. Wang et al. [33] evaluated the influence of graphene flakes in cement composite keeping w/c constant as 0.35 and GNPs/cement of 0.05%. The XRD patterns of control sample and cement paste with graphene flakes at 7 and 28 days are presented in Figure 15. The authors observed that no new phases were found after the incorporation of graphene in the cement mix. Hence, the type and structure of the final hydration products remained unchanged. However, the XRD spectra characterize the degree of the hydration process. Peak intensities of the hydrated cement products, i.e., calcium hydroxide and ettringite (AFt) were higher in graphene cement composite as compared with plain cement. The intensity of unhydrated cement content, i.e., alite (C_3_S) was also found to be lower in graphene cement composite. In addition to this, Murugan et al. [114] found that mineralogical composition also remained the same.

**Figure 16:**
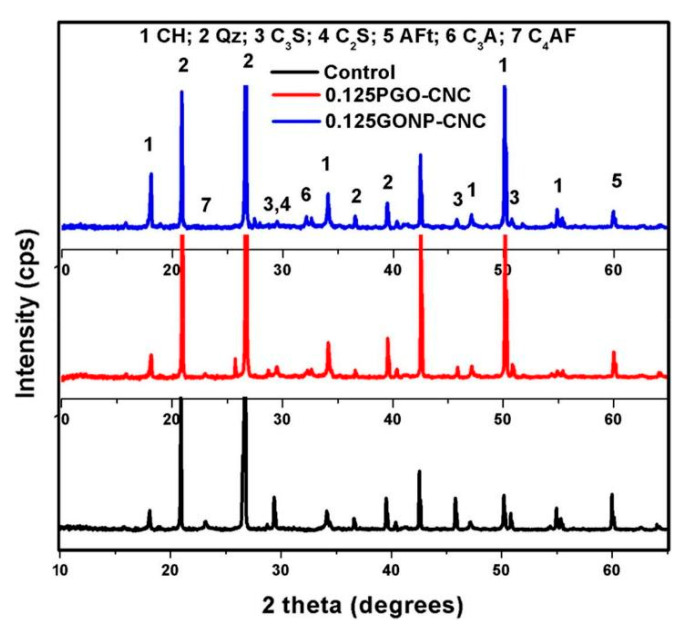
X-ray diffractograms of pristine graphene oxide mortar cement nanocomposite, graphene oxide nanoplatelets mortar composite and control samples. Reproducde with permission from [107], Elsevier, 2016.

The XRD patterns for control mix, 0.125 PGO-CNC, and 0.125 GONO-CNC after 90 days of curing were obtained by Sharma and Kothiyal [107] and presented in Figure 16. The quantity of portlandite was found to be greater with the incorporation of GO in cement-based composite. The authors found that peaks of C_3_S and C_2_S were reduced significantly by incorporating the graphene in the cement mix. Brittleness of composite mix was found less as compared with the plain sample because of the absence of Aft peak. It was suggested that the relative decrement of the C_4_AF (Tetra calcium aluminate ferrite) peak in the composite mix indicates the higher hydration rates due to nanocomposites.

### 5.4. Morphological Analysis

**Figure 17:**
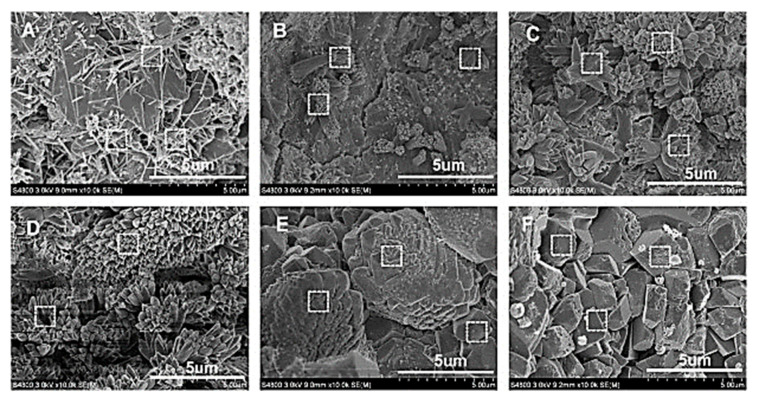
SEM images of cement composite at 28 days mixed with graphene oxide at various percentages: (**A**) no GO; (**B**) GO 0.01%; (**C**) 0.02%; (**D**) 0.03%; (**E**) 0.04%; and (**F**) 0.05%. Reproduced with permission from [115], Elsevier, 2013.

Various researchers have used field emission scanning electron microscopy (FESEM) to evaluate the morphology of cement-based composite prepared with graphene and its derivatives. The microstructure and pattern of hydrated crystals of graphene oxide based cement composite were investigated by Shenghua et al. [115]. The SEM of fractured surfaces are presented in Figure 17. The authors reported that disorderly stacked hydration products were formed in plain cement paste (Figure 17a). In contrast, with an increasing percentage of GO in cement composite, flower-like crystals as shown in Figure 17b,c) were formed. These flower-like crystals became denser with the rise in GO percentage up to 0.03% (Figure 17d). For a 0.04% dosage of GO, an irregular polyhedral resembling shape appeared in the hydrated products (Figure 17e), and for 0.05%, they became regular and complete polyhedral shapes (Figure 17f). According to authors, these flower-like crystals improved the toughness while polyhedron like crystals contributed to compressive strength. Shenghua et al. [115] concluded that GO regulated the cement hydration process and formed the regular flower-like and polyhedral like crystals. Furthermore, Shenghua et al. [115] evaluated the effect of hydration time for 0.03% of GO cement composite. According to the authors, GO contributes to the formation of the flower-like structure after 1 day of casting and on 28-day, these crystals became perfect and large flower-like shape. It was confirmed that graphene oxide regulated the hydration crystals in flower-like shape and these shapes tended to form a massive and compact structure through the cross-linking of flower-like crystals.

**Figure 18:**
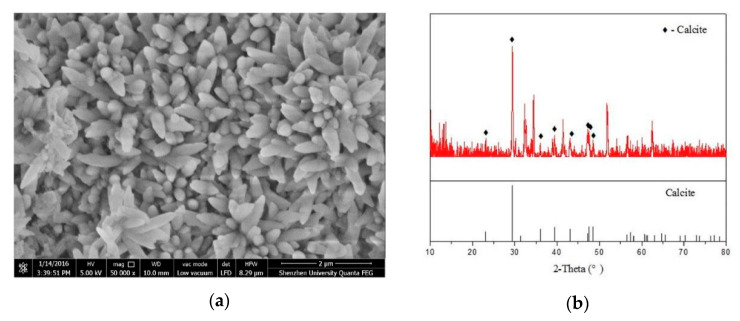
Description of the flow-like crystal (**a**) SEM image; and (**b**) XRD results of the surface of the sample obtained by method II. Reproduced with permission from [116], Elsevier, 2017.

Cui et al. [116] studied the chemical composition of flower-shaped crystal and polyhedral like hydrated crystal as reported in research of Shenghua et al. [115]. They proposed that these are calcium carbonates, which are due to the carbonation of cementitious hydrates and are not the product formed by cement hydration. Cui et al. [116] anticipated a potential pitfall in sample preparation for the scanning electron microscopy by Shenghua et al. [115], which resulted in the production of flower-like crystals. In order to prove this statement, Cui et al. [116] performed experimental work using carbon nanotubes with –COOH functional group. They collected two samples for SEM analysis from the same mother cube using two methods. For the method I, the obtained sample was oven dried for 24-h, after which, the sample was cooled down to room temperature for SEM analysis. For method II, the obtained sample was placed in a natural environment for seven days. After that, sample was oven dried and then cooled for SEM analysis. Figure 18 showed the SEM image and XRD pattern of the sample obtained from method II. The calcite peaks were distinguished and prominent in Figure 18b. Thus, Cui et al. [116] recommended further research on the regulation mechanism of GO on cement hydration as suggested by Shenghua et al. [115].

**Figure 19:**
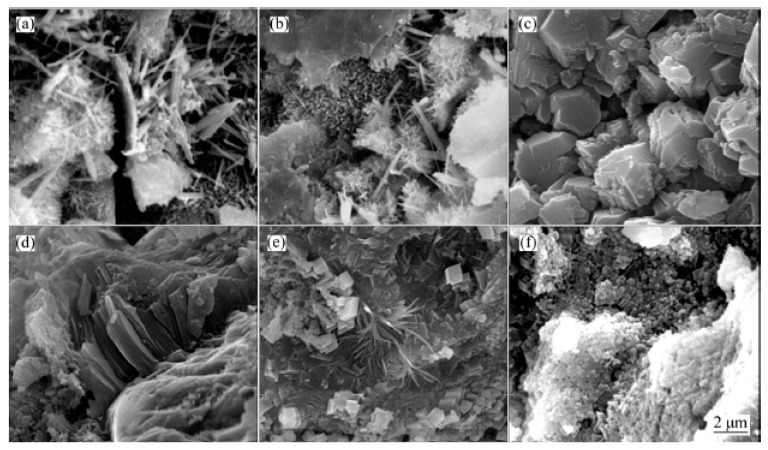
The effect of functionalized FGN to the cement composite after 28 days with varying FGN content (**a**) No FGN; (**b**) 0.01% FGN; (**c**) 0.02% FGN; (**d**) 0.03% FGN; (**e**) 0.04 % FGN and (**f**) 0.05% FGN. Same scale bar was used in (**a**–**f**). Reproduced with permission from [117], Springer, 2016.

Cao et al. [117] stated that the structure of the hydrated cement products showed disorderly production of needle-shaped ettringite and hexagonal calcium hydroxide as shown in Figure 19a. After the addition of functionalized graphene nanosheets (FGN) in mix design, the structure of hydrated cement products became more compact and less needle-shaped crystals (Figure 19b). When FGN percentage increased to 0.02%, it formed the polyhedron shape (Figure 19c) which represents the compact structure. However, the high content of functionalized graphene nanosheets (0.03% to 0.05%) led to the decrease in degree of hydration product because of the attachment of hydrophilic groups on the surface of FGN, which absorbed some part of the available water and prevented the full hydration process of cement pastes (Figure 19d–f) [117].

Therefore, it can be concluded form a literature of microscale characterization that no chemical interaction and new phase formation was observed in graphene cement composite. However, graphene-based nanomaterials act as accelerator and dense formation of hydrated cement product was found near the nanomaterials. A detailed and in-depth study is still required to completely understand and explore the influence of graphene-based nanomaterials on hydrated cement products at the microscale level.

## 6. Rheological Properties of Graphene Cement Paste

Concrete possesses great advantages which makes it the most extensively used building material [118]. During mixing and placing of concrete, key properties are determined to be fluidity, homogeneity, consistency, and workability [118]. Any deficiencies in these properties are prone to contribute to laitance, segregation, bleeding and cracking of the concrete [119]. It is known that the mechanical and durability properties of cementitious construction components depend on viscosity and fresh properties of cement composite. Any variation in viscosity may lead to defects in concrete structures [120]. Hence, the rheological properties of concrete are extremely significant to attain homogeneity and obtain improved workability. The rheology of cement paste firmly impacts the overall fresh properties of concrete [121]. Typically, shear stress and shear rate are the indicators of the flow properties of the cement paste. Next, using flow curves and mathematical models, viscosity and other flow parameters are determined. With the improvement in nanotechnology, researchers are now focusing more on evaluating the influence of nanomaterials on cement composite [122].

**Figure 20:**
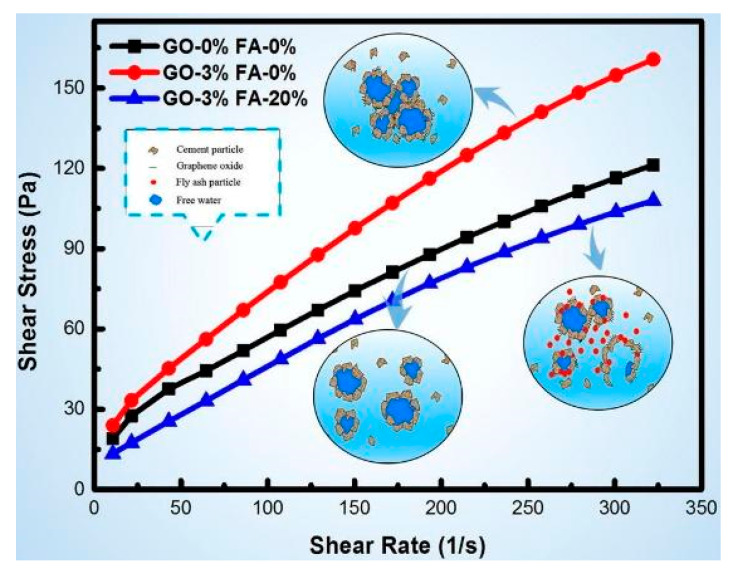
Impact of fly ash on flow curves of GO cement paste. Reproduced with permission from [127], Elsevier, 2017.

Many researchers examined the influence of different nanomaterials on cement paste flow properties [123,124,125,126,127,128]. Ormbsy et al. [123] used the parallel plate geometry for rheological investigation and found that MWCNTs meaningfully influenced the rheological behaviour of polymerizing cement. Konsta et al. [47] used four different length of MWCNTs while the surfactant to MWCNTs weight ratio was kept as 1.5, 4.0, 5.0, and 6.25. In the aqueous solution, the MWCNTs content was kept constant at an amount of 0.16% by weight of water. The MWCNTs were treated using with and without sonicated energy. After that, MWCNTs aqueous-surfactant suspensions were mixed with cement and rheological properties were investigated. In preparation of MWCNTs cement composite, MWCNTs were used with the content of 0.08% of cement (by weight) and w/c = 0.5. Preliminary rheological results indicated that long MWCNTs are difficult to disperse. They observed the shear thinning response of cement paste and at high shear stress (above 70 Pa), approximately constant viscosity independent of sonication energy was obtained. Shang et al. [125] investigated the rheological properties of GO and GO encapsulated silica fume-based cement pastes using Bingham model. The authors found that GO reduced the fluidity of the cement paste by 36.2% when compared to control cement paste. A rise in the value of yield stress and plastic viscosity was obtained when GO was added to the cement paste. Wang et al. [126] mentioned that the addition of GO to cement paste forms flocculation particles, which had a dependence on the GO concentration and it, consequently, improved the yield stress, plastic viscosity, and area of the hysteresis loop of the flow curve. Also, they investigated the effect of fly ash on the GO cement paste and found that for 0.01 wt.% of GO and 20 wt.% of fly ash, the yield stress and plastic viscosity of the cement paste dropped when comparing with control cement paste by 85.81% and 29.53%, respectively. For cement specimen with 0.03 wt.% of GO and same amount of fly ash, the yield stress and plastic viscosity of the cement paste was lowered by 50.33% and 5.58%, respectively (Figure 20).

Yahia et al. [129] found that estimated viscosity and yield stress values varies according to the mathematical model used. Various researchers used different mathematical models to determine the yield stress and plastic viscosity values and predicted the specific tendency of the flow. Due to statistical errors [130], it is impossible for one model to predict precisely the trend of the flow behaviour of cement paste [129].

**Figure 21:**
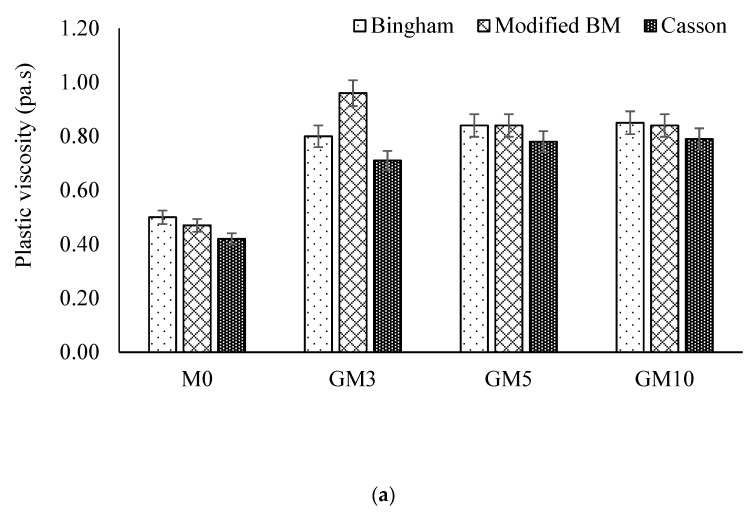

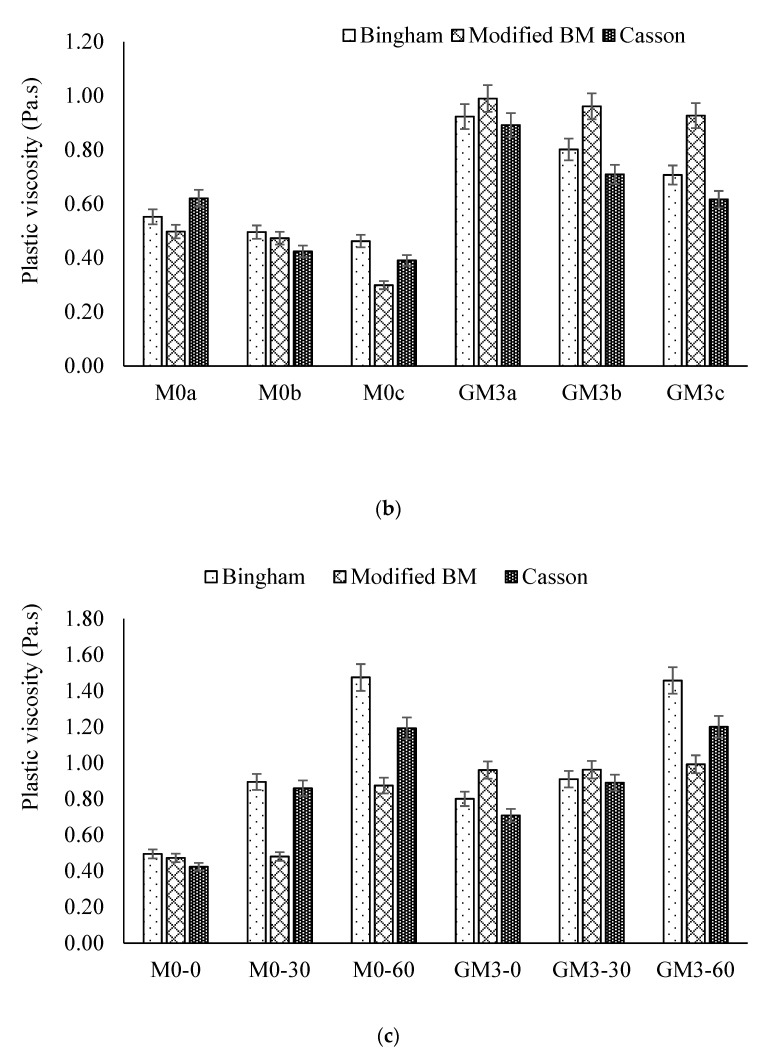
Plastic viscosity values (Pa·s) calculated by various mathematical models with varying (**a**) GNP amount, (**b**) shear rate and (**c**) resting time. Reproduced with permission from [118], MDPI, 2017.

The rheological properties of fresh cement paste with different surface area of GNPs, resting time and shear rate cycles were examined by Rehman et al. [118,131] using various rheological mathematical models. The authors observed the increase in plastic viscosity and yield stress with the increase in intensity of graphene in the composite and resting time but a decrease was noted for higher shear rate cycle. Furthermore, it was found that the highest values of yield stress were obtained when measured by the modified Bingham model (BM) and the lowest values were estimated by the Casson model. Figure 21 shows the plastic viscosity values for graphene cement composite and the influence of different parameters on it. It was concluded that the modified BM fits the experimental flow curves best and the Casson model demonstrated greater standard error values.

It can be concluded from the existing available literature that new mathematical models need to be developed which will predict the rheological properties of nanomaterial-based cement composite. Moreover, the influence of graphene-based nanomaterials requires further exploration so that it can be successfully used in 3D printing applications of the construction industry.

## 7. Mechanical Properties of Graphene Cement Composite

**Figure 22:**
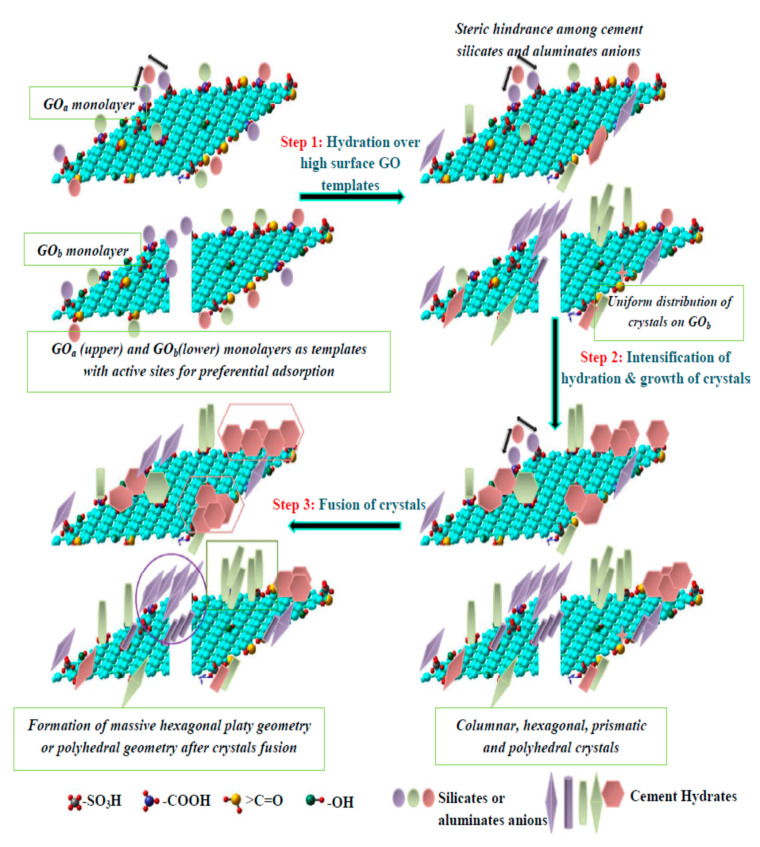
Schematic diagram of growing cement hydrates on templates of GO monolayers. Reproduced with permission from [138], RSC Advances, 2015.

Consumption of cementitious building materials i.e., concrete is increasing due to its various advantageous characteristics such as high strength, durability, and resistance to fire [2]. Sixuan [79] conducted a study on the mechanical properties of both cement paste and mortar by adding a different concentration of GNPs. Their study revealed that there was no increment in compressive strength for the cement paste incorporated with 0.05% and 0.25% of GNPs. However, an increment of 20% in compressive strength was observed for 0.50% of GNP added to cement mortar. As for the flexural strength, the maximum increment up to 82% was observed for the cement paste with 0.05% of GNP [79]. Table 3 provides the effect of graphene nanomaterials and its derivatives on the mechanical properties of cement composite. The incorporation of nanomaterials in cement composite significantly increased the mechanical properties of cement composite both at early and later age. Wengui et al. [132] proposed that due to the nucleation and filling effect of GO, it can speed-up the hydration process at an early stage. Yet, the complete mechanisms have not been described in the available literature. For example, the compressive and flexural strength reported by Shenghua [133] and Mokhtar et al. [134] are considerably different. Both researchers used the same water-to-binder ratio (w/b), the GO dosage and almost similar curing conditions. Moreover, Wang et al. [127] and Wang et al. [135] used the same w/b and GO dosage yet the difference in the rise of compressive strength was almost double. Under similar conditions, the increase in compressive strength reported by Kothiyal et al. [136] and Shang et al. [125] is twice as that reported by Sharma and Kothiyal [107] and Wengui et al. [132] respectively. It is commonly known that the factors influencing the mechanical strength in the composite matrix are w/b, type of nanomaterial, its dosage and curing duration of the specimen [137]. Moreover, the results of dispersion, agglomeration, size, and functional groups attached with nanomaterials have considerable influence on mechanical properties [28]. It can be observed from Table 3 that the nanomaterials have more influence on the flexural strength of cement composite as compared with compressive strength. According to Sharma and Kothiyal [138], the bridging and bonding effect of GO with cement matrix and dense microstructure of cement matrix are the factors contributing to flexural strength. Figure 22 shows the schematic of growth of cement hydrates on the templates of GO. Furthermore, the use of dispersant, surface modification, reduction in size and thickness of graphene has been employed to improve the performance of GNDs on mechanical properties of cement-based materials [138,139,140]. It is understood that the key factors controlling the porosity and the mechanical properties of cement-based materials are the availability of more hydrated cement products, filling of pores and bonding between hydrated cement products and nanomaterial [138,141,142]. Nevertheless, the tremendous increment in the mechanical properties has been recorded, yet the role of graphene nanomaterial is still unclear in the literature.

Enhanced mechanical properties of graphene cement composite are attributed mainly to the bonding of graphene and its functional groups with CSH gel [138]. Hou et al. [143] performed simulation work using ReaxFF forced field to explore the molecular-scale structure and chemical interaction between the functional groups of graphene and CSH. In order to strength the simulation results, Hou et al. [143] also performed the experimental investigation. The experimental results showed that by incorporation of 0.16% GO by weight of cement in GO cement composite, the compressive and flexural strength enhanced by 3.21% an 11.62% respectively. For further detailed analysis, the mechanical behaviour of G_CSH, GO_CSH and GO_CASH models under tension loading was studied using a stress–strain curve. They found that graphene without chemical bonding with CSH in G_CSH model showed a small increase in compressive stress. The authors concluded that functionalization significantly enhanced the mechanical properties due to interfacial strength between functionalized GO and CSH gel. In addition, weak bonding and instability of atoms in the interface region resulted in the weakest mechanical behaviour.

## 8. Energy Harvesting and Thermoelectrical Properties of Graphene Cement Composite

**Figure 23:**
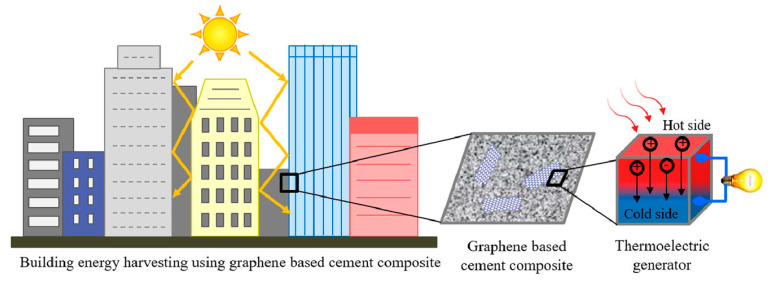
Building energy harvesting using thermoelectric generator of graphene cement composite. Reproduced with permission from [164], Elsevier, 2019.

Energy harvesting is a concept through which existing ambient energy in the environment e.g., solar, wind, hydro energy has been converted into useful forms i.e., electrical energy [163]. According to Francisco and Adelino [163], micro energy harvesting, which is associated with small scale energy harvesting is gaining interest in the scientific community. In micro energy harvesting, the main energy sources are heat, thermal variations, acoustic emissions, electromagnetic variations and mechanical vibrations. In this section, the application of graphene cement composite for energy harvesting using its thermoelectrical properties will be discussed. A schematic of energy harvesting in buildings by converting the abundant solar energy into electrical energy is demonstrated in Figure 23. Graphene cement composite have been used by researches for energy harvesting in buildings by enhancing the thermoelectrical properties of cement composite [164]. Thermoelectrical properties are measured in terms of dimensionless figure of merit, ZT, which is equal to (S^2^σT/κ), where S is Seebeck coefficient in µVK^−1^, σ is electrical conductivity in Scm^−1^, T temperature in K and thermal conductivity κ in Wm^−1^K^−1^ respectively. In addition, ZT ≥ 1 is recommended for energy harvesting application purposes in buildings. Moreover, graphene has the capability to transform the non-conductive material into conductive material. According to Balandin [165], graphene enhanced the electrical conductive properties of cement composite and reduced the thermal conductive properties.

**Figure 24:**
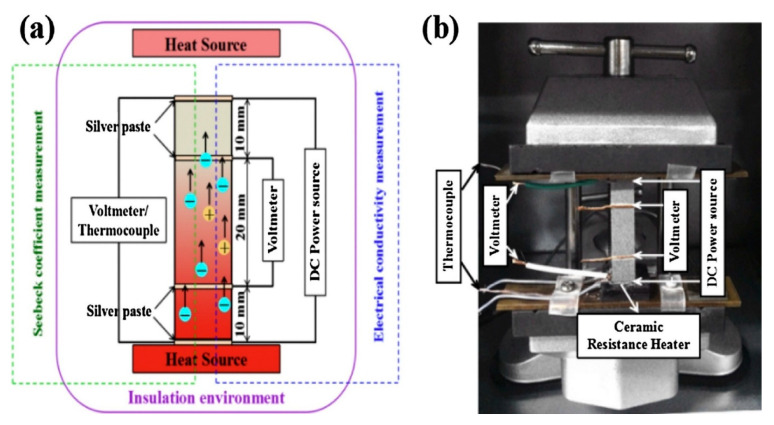
Laboratory setup for evaluating the seebeck coefficient and electrical conductivity (**a**) The test principle diagram and, (**b**) the experimental setup. Reproduced with permission from [166], Elsevier, 2018.

It was Wei et al. [166], who for the first time measured the thermoelectrical properties of expanded graphite cement composite for large scale energy harvesting and climate adaptation. Expanded graphite was added as 5, 10 and 15 by mass of cement for the preparing the graphite cement composite. Figure 24 shows experimental setup for determining the thermoelectrical properties of graphite cement composite. The authors found that ZT values depend on the temperature. The values of ZT increased from 30–75 °C while it decreased from 75–100 °C. The maximum ZT value of 6.82 × 10^−4^ was noted for 15% addition of graphite in cement composite at 75 °C. Overall, ZT values were found to be smaller due to lower electrical conductivity and seebeck coefficient of graphite cement composite. However, due to large areas of graphite cement composite in pavements and roofs being exposed to solar radiation, the solar energy can be converted into electrical energy during the summer season around the globe. Table 4 presents the thermoelectrical properties of graphene based cement composite determined by various researchers.

**Figure 25:**
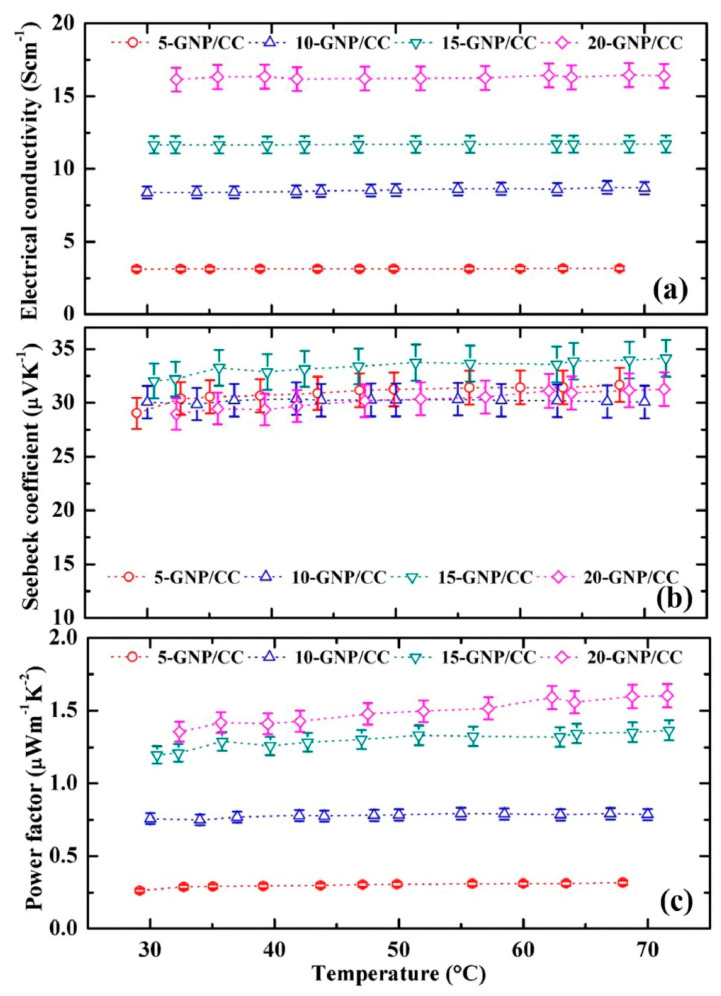
Estimation of (**a**) electrical conductivity, (**b**) seebeck coefficient and (**c**) power factor of graphene cement composite. Reproduced with permission from [164], Elsevier, 2019.

Ghosh et al. [164] performed experimental study using graphene nanoplatelets in cement composite to determine the thermoelectrical properties and energy harvesting capability of composite material. They employed GNP as 5%, 10%, 15%, and 20% by mass of cement to prepare graphene cement composite. Four-probe electrical conductivity and seebeck coefficient measurement system was used to determine the Seebeck coefficient and electrical conductivity simultaneously. For testing, rectangular specimens were used and experiment was performed with varying temperature from 25 °C to 75 °C with heating rate of 0.01 °C/s. Furthermore, differential scanning calorimeter (DSC) was used by the authors to monitor the specific heat capacities within the temperature range of 25 °C to 75 °C. The authors observed largest seebeck coefficient of 34 µVK^−1^ for 15% GNP inclusion at 70 °C, while electrical conductivity of 16.2 Scm^−1^ and power factor of 1.6 µWm^−1^K^−2^ for 20% GNP inclusion as shown in Figure 25. The linear relationship between temperature and power factor was also observed. Highest specific heat capacity value 0.88 Jg^−1^K^−1^ was found for 10% GNP cement composite while 20% graphene cement composite showed highest thermal diffusivity as compared to other percentages of graphene cement composites. Moreover, the highest ZT of 0.44 × 10^−3^ was found for the 15% GNP cement composite at 70 °C. According to authors, the higher amount of GNP in the cement composite was beneficial for establishing the conductive network. It was concluded that graphene based cement composite significantly contribute to energy harvesting application in addition to improving the quality of indoor environment of buildings. With this application, graphene cement composite can harvest the energy, reduce electric consumption, and provide a substantial financial benefit [164].

## 9. Piezoresistive Properties of Graphene Cement Composite

Nanomaterials are gifted with the amazing characteristics and their incorporation in cement composite improved the properties of nanomaterial based cement composite. The reason for the presence of electrical properties of graphene is the half-filled band that permits free-movement of electrons. The π-bonds hybridize together to form the π-band and π*-band, which are responsible for the electrical properties of graphene [169]. Self-sensing and damage memorizing capability of graphene-based cement composite are one of the characteristics, which graphene brought in cement composite.

The electrical properties of the cement composites are also of great importance and may be used to control damage in a concrete structure. The cement-based composite reinforced with conducting fillers can recognise its own strain by indicating the variations in the electrical resistivity values. According to Rehman et al. [118], the piezo-resistive properties are the result of a change in electrical resistance in the specimen subjected to mechanical strain [170]. Hence, electrical resistance of the cement composite is measured. By definition, the electrical resistance is the strength of a material in opposing the electrical current flowing through it. Researchers have mostly used the four-probe method to investigate the electoral properties of cement composite. In four-probe method, voltage is measured using the inner two electrical contacts while the current is measured using the outer two electrical contacts [171]. In comparison with the two-probe method, the four-probe technique is better since the calculated resistance does not include contact resistance [172].

Han et al. [172] reported that the distance between the current and voltage poles has a great importance but its impact is insignificant if the space is larger than 7.5 mm. Numerous researchers performed experiments with various spacing values. For instance, Geng et al. [173] used 10 mm while Liang et al. [174] used 40 mm distance between voltage measuring probes and current. Geng et al. [173] used the 40-mm gap between two measuring probes while Liang et al. [174] used an 80-mm gap. For unequal spacing, the electrical resistivity values are calculated by using Equation (1) [175].

(1) $$\rho \;=\;\frac{V}{I}\;˟\;2𝜋\;\;˟\;\frac{1}{\;\left(\frac{1}{S1}+\frac{1}{S3}-\frac{1}{S1+S2}-\frac{1}{S2+S3}\right)}$$

where, *S*_1_, *S*_2_, and *S*_3_ are the spacing in cm and calculated from current carrying probe to the voltage measuring probe. Various researchers used the piezoresistive characteristics of nanomaterials based cement composite for self-sensing prose [173,176,177]. Hence, the piezoresistive properties of nanomaterial-based cement composite are critically reviewed in the following paragraphs.

According to Hui et al. [178], the self-sensing property of the nanomaterials with cement composite may be used for structural health monitoring (SHM) purposes with no need of any additionally attached or embedded sensors. It will further open the application of nanomaterials based cement composites for fabricating smart sensors [178]. Geng et al. [173] conducted research on the functionalization of CNTs using carboxyl. The effect of functionalization of CNTs on the piezoresistive and electrical properties of the cement composite was observed. It was noted that the electrical resistance of cement composite with 0.5% of carboxyl functionalized CNTs (SPCNT) was 149 Ω.cm and for plain CNTs (PCNT) was 130 Ω.cm. Thereafter, the fractional change in electric resistivity for both types of cement composited was recorded against the cyclic compressive loading. The results showed that both cement composites were capable of monitoring the applied compressive cyclic loading, however, SPCNT specimen showed better response as compared with PCNT.

**Figure 26:**
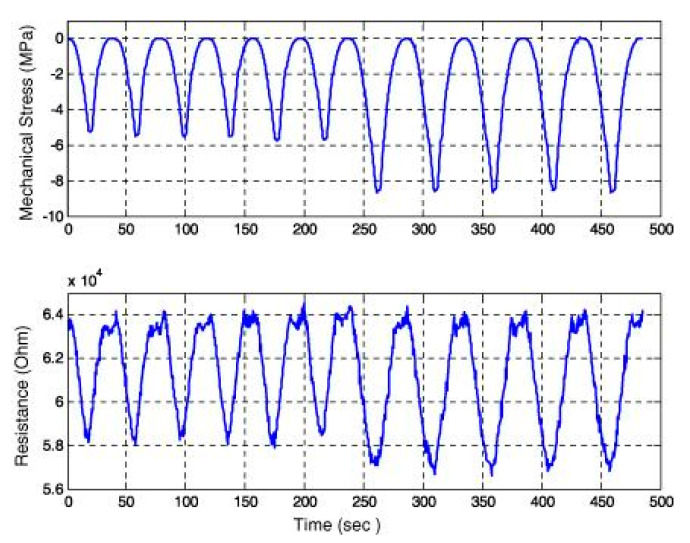
The piezo resistivity behaviour of cement paste enhanced with 0.1% of carboxyl functionalized MWCNTs. Reproduced with permission from [179], IOP Sciences, 2009.

Xun and Kwon [179] studied the piezoresistive behavior of the CNT based cement composite and investigated its potential application as an embedded sensor in civil infrastructure. It was shown that the electrical resistance of CNT-based cement composite was following the same trend as applied compressive loading (Figure 26). The authors proposed that the fabrication method needs to be further optimized and the response of these composite materials must be investigated in concrete. Furthermore, they proposed that if these composite stress sensors were embedded in civil infrastructure, e.g., pavements or bridges, then due to their compatibility with concrete, they will have a long service life with the least maintenance.

**Figure 27:**
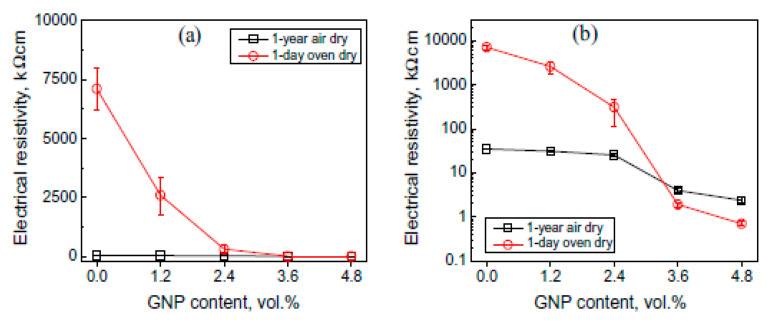
The relation between the electrical resistivity of the GNPs cement mortar composite and its respective GNPs content in (**a**) linear scale and (**b**) log scale. Reproduced with permission from [174], Elsevier, 2014.

The influence of different amounts of GNPs on the sensing behaviour of the cementitious composite was investigated by Hingiian and Pang [180]. The authors noted a decrease in the electrical resistivity of cement composite when GNPs was increased from 2.4% to 3.6%. Liang et al. [174] investigated the electrical resistivity values of cement mortar with increasing content of graphene flakes. For this purpose, the specimen was subjected to two different environmental conditions, i.e., one specimen was air-dried for one year, and another specimen was oven-dried at 1-day after casting. The authors proposed a link between the electrical resistivity values of the specimen with increasing content of GNP as presented in Figure 27. The electrical resistivity values decreased as GNPs content increased. 2.4% of GNP was estimated as the percolation threshold value for GNP-composite mortar [174]. The decrement of more than one order occurred when GNP was increased from 2.4% to 3.6% in cement mortar, as shown in Figure 27b.

**Figure 28:**
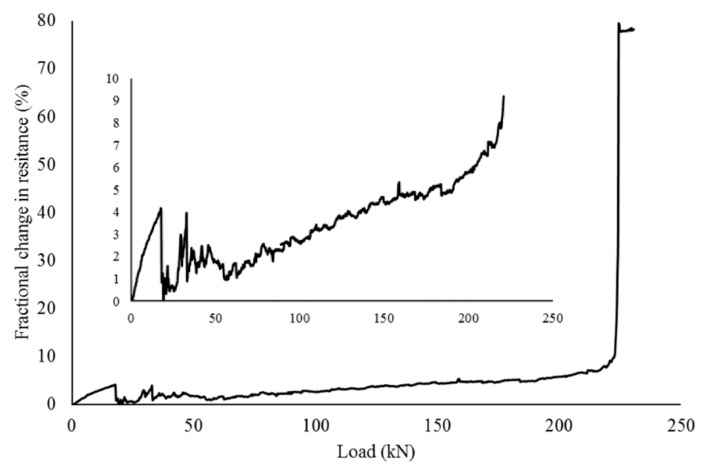
Fractional change in resistance of graphene cement smart sensor specimen subjected to compressive loading on the reinforced concrete beam. Reproduced with permission from [118], MDPI, 2017.

Rehman et al. [118] investigated the application of graphene cement smart sensor in a GNPs reinforced concrete beam. This beam was subjected to flexural loading and the values of fractional change in resistance (FCR) of graphene cement composite specimen were recorded as shown in Figure 28. The authors observed that FCR values varied as the applied loading on the beam increased and a sudden response was indicated when the beam failed. The conclusion of the experiment was that the cement composite containing graphene favourably responded against crack propagation. The authors concluded that the graphene cement composite specimen is an inexpensive and effective way to control the structural health of the members during its shelf life.

As existing well-known methods of health monitoring have some limitations in the application [9] and most importantly, these methods require additional cost for health monitoring purposes. Therefore, it is required that smart sensors with self-sensing characteristics should be developed. These smart sensors are required to reduce health monitoring costs and make construction projects financially more economical. Furthermore, they should be compatible with the cement-based building materials and should have the capability to be used for health monitoring purpose.

## 10. Discussion and Research Gaps

In the literature, various nomenclature has been used which could create confusion for the readers. Therefore, a detailed description of graphene and its derivation including its precursors is provided in Table 5.

It has been noted that GNPs enhance the hydrated cement products at an early age of 7-days in contrast to 28-days. Furthermore, no chemical interaction and new phase formation in hydrated cement product took place in the production of GNPs and hydrated cement products. However, from infrared spectral analysis Li et al. [48] found that a chemical interaction exist between hydrated cement product and functional groups (hydroxyl and carboxyl) of carbon nanotubes. The variation in CSH phases was also monitored. Some researchers like Murugan et al. [114] did not find any major difference in IR spectra. Several researchers [33,158] also noted that the type and structure of hydrated cement products remained undisturbed. Yet, they noted that GNPs acted as accelerator in the hydration process and enhanced early age strength. Some researchers like Alkhateb et al. [32] found high density CSH near GNPs using SEM images and concluded that high interfacial strength is available between graphene and CSH gel. Shenghua et al. [115] observed the generation of flower-shaped and polyhedral-like crystals on GO sheets and concluded that GO regulates these hydration crystals. However, Cui et al. [116] investigated the chemical composition of these flower like crystals and noted that these crystal formed due to carbonation of hydrated cementitious products not by the graphene oxide. Various researchers have also stated that due to nucleation and filling effect of graphene, early age hydration process accelerated. Yet, the complete mechanism has not been explained. Graphene and its derivatives also influenced the rheological behaviour of cement composite. According to Wang et al. [126] and Rehman et al. [118] these nanoparticles form the flocculated structures and entrapped the water molecules. However, the trend of flow behaviour of a composite material significantly depends on the mathematical model and its assumptions. Mechanical properties of cement-based composite are also important. Due to nucleation and filling effect of graphene, it has been found that the mechanical properties enhance significantly. Bridging and blockage in crack propagation at nanoscale level also contribute to and influence the mechanical properties of cement-based composite. The molecular modelling approach suggest that good bonding and stability of atoms in interface region play an important role for enhancing the mechanical properties of graphene cement composite. Furthermore, energy harvesting and the thermoelectrical properties of graphene cement composite will benefit the socioeconomic system and reduce the electricity consumption in buildings. Some incredible characteristics of graphene like self-sensing, crack monitoring and damage memorization properties outshine it. Four probe electrical resistivity method has been utilized by various researchers for self-sensing characteristics. Spacing between current pole and voltage pole is important for development of sensors to be used for health monitoring of concrete structures. Various researchers found fractional changes in resistance against the cyclic compressive load and this has opened the gateway for the use of these sensors in the highway and transportation industry.

It was found that the graphene based nanomaterials have several issues which, needs to be resolved before its usage in the construction industry. The following research gaps have been identified.

(1) Very limited research has been found regarding the manufacture of nano-size cement particles and nano-binders however, it has great potential for the development of novel admixtures, nanoparticles, and nano-reinforcements.
(2) Most of the researches on the dispersion of graphene and its derivatives were focused on surface modification, functionalization, and oxidation process. However, these processes damaged the atomic structure of graphene. Therefore, other methods are indispensably required for the dispersion of GNDs and preserving the atomic structure of graphene.
(3) The enhancement mechanism in properties is not completely described as yet. Further research is required to study the regulating mechanism of graphene and its derivatives on hydrated cement crystals.
(4) Flow properties of graphene cement composite and its dependence on various factors are missing in the existing literature. The variation of geometric flow with time, dispersing agent, shear rate and various types of graphene sheets is required to explore the flow of cement paste in the plastic state. Moreover, the best optimized rheological mathematical model needs to be sorted out, as a single rheological model cannot predict the flow behaviour of cement paste accurately. Thus, graphene cement composite demands further exploration to achieve maximum benefit from graphene.
(5) The complete mechanism for accelerating the hydration reactions and enhancing overall mechanical properties has not been explained. Prominent differences in mechanical properties were found in experimental work conducted by various researchers under similar experimental conditions.
(6) The distance between the current and voltage poles is required to be optimized for four-probe method. The potential application of graphene-based cement composite as an embedded smart sensor in the real concrete structure was seldom found in the literature. Moreover, the suitability, industrial demand, and compatibility of graphene cement smart sensors with the existing non-destructive health monitoring methods need to be determined.

## 11. Conclusions

In this review paper, we have critically reviewed recent research on graphene based cement composites. It was demonstrated that dispersion of graphene is critical for its effective utilization in cement based composites. Several characterization techniques, such as TGA, IR, XRD, and SEM, were discussed in detail to evaluate the impact of graphene on the performance of cement-based composites. Conclusively, graphene was found to enhance the rheological, mechanical, thermoelectrical, piezoresistive, self-sensing, and damage memorizing capabilities of cement-based composites.

## Figures and Tables

**Table 1:** Peak (C=C bonds) of UV-vis absorbance of rGO from various reduction approaches.

π-π* Transition of rGO	References
275 nm	[89]
273 nm	[90]
273 nm	[89]
272 nm	[91]
271 nm	[92]
271 nm	[93]
270.9 nm	[94]
270 nm	[95]
269 nm	[96]
269 nm	[97]
269 nm	[98]
267 nm	[99]
266 nm	[100]
265 nm	[101]
264 nm	[102]
263 nm	[103]
261 nm	[104]
260 nm	[105]

**Table 2:** Observed peak values in FTIR spectra of control sample. Adapted from [48].

Bond Type	Wavelength (cm^−1^)
H-O-H stretching of CSH	3375
Si-O asymmetric stretching vibrations of CSH	1014
Si-O in-plane vibration of CSH	460
Si-O out of plane vibration of CSH	690
C-O Stretching of CO_3_^−2^	1410
C-O Stretching of CO_3_^−2^	874
C-O Stretching of CO_3_^−2^	712
H-O-H stretching of ettringite	1630
H-O-H stretching of ettringite	3430
S-O bending vibration of SO_4_^−2^	695
C=O, C=C, O=O	2299
C=O, C=C, O=O	2075

**Table 3:** The impact of graphene based nanomaterials on the mechanical properties of cement composite modified from [28].

Matrix	Nanomaterial Type/Dosage (wt.%)	w/b	Percentage Rise in Compressive Strength/Age	Percentage Rise in Flexural Strength/Age	Reference
Cement Paste specimens	GO/0.02	0.3	13.0/28 d	41.0/28 d	[134]
			60.1/28 d	84.5/28 d	[133]
	rGO/0.02		22.0/28 d	70.0/7 d	[114]
	GO/0.03		18.8/28 d	56.6/28 d	[135]
			42.5/28 d	55.0/28 d	[127]
	FGON/0.03		51.3/28 d	65.5/28 d	[140]]
	GO/0.04		28.6/28 d	43.2/28 d	[144]
	GO/0.05		66.4/7 d	69.4/7 d	[145]
			52.4/3 d	90.5/28 d	[146]
	GNPs/0.15		49.4/28 d	27.5/28 d	[147]
	GO/0.022	0.4	27.6/3 d	26.7/3 d	[148]
	GNPs/0.03		1.3/28 d	16.8/28 d	[33]
			30/28d d	-	[118]
	GO/0.04		37.0/28 d	14.2/28 d	[132]
			15.1/28 d	–	[125]
	GO/0.05		11.0/15 d	16.2/15 d	[149]
	GO-CNT/0.05		21.1/15 d	24.1/15 d	[149]
	GO-CNFs/0.05		2.89/28 d	25.0/28 d	[150]
	GO-CNT/0.06		23.9/28 d	16.7/28 d	[151]
	GO/0.03	0.5	40.0/28 d	–	[152]
Cement Mortar Specimens	GO/0.022	0.4	–	34.1/7 d	[153]
	GO/0.03		–	18.7/7 d	[154]
			45.1/3 d	70.7/3 d	[155]
	GO/0.05		43.2/3 d	106.4/14 d	[146]
	GO/0.02	0.5	–	36.7/3 d	[156]
	GO/0.03		30.0/28 d	–	[157]
	FGON/0.03		20.3/28 d	32.0/28 d	[158]
	FGON/0.1		39.0/15 d	70.8/15 d	[139]
	GNPs/0.1		19.9/28 d	–	[159]
	GO/0.125		110.7/3 d	–	[136]
			53.0/3 d	–	[107]
	GNPs/0.8		87.5/28 d	–	[160]
	GO/1.0		114.1/14 d	–	[138]
	GNPs/0.08	0.6	55.3/7 d	–	[161]
Concrete	GO/0.1	0.5	14.2/7 d	4.0/3 d	[162]

**Table 4:** Thermoelectrical properties of graphene based cement composite.

Materials	Concentration (wt.%)	S (µV/°C)	σ (Scm^−1^)	*K* (Wm^−1^K^−1^)	Power Factor (µWm^−1^K^−2^)	Reference
Control sample without nanomaterial	0	10^−5^–10^−4^	10^−7^	0.53		[166]
Expanded Graphite	5	−54.5	0.2	1.619	0.1	[166]
	10	−51.5	7.4	2.594	1.9	[166]
	15	−50.1	24.8	3.213	6.38	[166]
Carbon nanotubes	15	57.98	0.818	0.947	-	[167]
Graphene nanoplatelets	5	32	3.13	0.743	0.4	[164]
	10	30	8.5	0.947	0.7	[164]
	15	34	11.68	1.067	1.25	[164]
	20	31	16.2	1.327	1.6	[164]
n-doped CNT (before drying)	1	−500	0.0173	-	0.435	[168]
n-doped CNT (after drying)	1	−58	0.0219	-	0.007	[168]
p-doped CNT (before drying)	1	−112	0.0054	-	0.009	[168]
n-doped CNT (after drying)	1	20	0.0069	-	0.0002	[168]

**Table 5:** Description of Graphene and its derivatives.

Sr. No.	Nomenclature	Description	Reference
Nanomaterials			
1.	Graphene	Graphene is single layer of densely packed carbon atoms in benzene- ring structure in 2-dimensions (2D). sp2 interacted carbon atoms are conned tightly and forms honeycomb lattice.	[88]
2.	Rolled sheets of graphene (CNT)	Carbon nanotubes (CNT) consist of sp2 carbon atoms arranged in honeycomb lattice in 1-dimension (1D) and capped with fullerene-like hemisphere at each end. They are also conceptualized as 1D rolled sheets of graphene.	[181,182]
3.	Graphene Oxide (GO);	Graphene Oxide (GO) is derived from graphene sheets and known as oxidized form of graphene. A mixture of carboxyl, hydroxyl and epoxide groups are attached chemically (covalent linkage) with graphene sheets.	[21,55,183]
4.	Graphite oxide,	Graphite oxide is identical chemically to Graphene oxide, However, inter-planar spacing between them vary due to oxidation process.	[184,185]
5.	Functionalized Graphene	Attaching various functional groups like epoxide, hydroxyl and carboxyl group with Graphene through covalent linkage is known as Functionalized graphene.	[21]
6.	Graphite/Three- dimensional graphite/Graphite structure/ Expanded graphite structure	3-D carbon allotrope containing minute crystallite of graphite. It is made of stacked graphene sheets with 0.345 nm spacing.	[186,187,188]
7.	2D flat sheet of carbon nanomaterial	Single layer graphene sheet is also known as 2D flat sheet of carbon nanomaterial.	
8.	Pristine graphene oxide (PGO)	Graphene oxide in its original condition (i.e., Ideal) and does not have a single defect.	
9.	Reduced graphene oxide (RGO)	Reduced graphene oxide (RGO) are prepared from oxidation, exfoliation and chemical reduction of graphite oxide.	[189]
10.	Reduced graphite oxide (rGO)	Main difference between reduced graphene oxide and Reduced graphite oxide is inter-planer spacing between atomic layers of the compound.	[184,185]
11.	Graphene and its derivatives (GNDs)	Various structural form of graphitic structure in 0D (carbon materials), 1D (CNTs), 2D (graphene sheets) and 3D (Naturally available graphite).	
Flakes			
12.	Graphene flakes (GNFs)	Graphene flakes are small tiny particles, which are much easier to form and handle in solution and powder form as compared with large graphene sheets. These GNFs possess some of the properties of large graphene sheets however; they vary with shape and size of tiny particles. Furthermore, maintaining uniform consistency in shape and sizes is not easy during synthesis process. Graphene flakes (GNFs) are also labelled as graphene nanoplatelets (GNPs).	[190]
13.	Graphene oxide flakes	Formation and production of graphene oxides in small tiny particles in powder or solution form is known as Graphene Oxide flakes.	[190]
14.	Exfoliated graphene flakes	Exfoliated large size graphene flakes/particles/sheets	
Nanoplatelets			
15.	Graphene Nanoplatelets (GNPs)	Graphene Nanoplatelets (GNPs) are small rounded disk-shaped tiny particles, However, it is difficult to uniformly produce them even if synthesized artificially. Theoretically, all GNPs are not disk-shaped particles, therefore should be considered as Graphene flakes (GNFs).	[191]
16.	Graphite Nanoplatelets (GnPs or GPs)	Graphite Nanoplatelets are composed of mixture of graphene layers with thicker graphite particles. Their chemical structure is in-between graphene and graphite. Some researchers also consider them as graphene nanoplatelets, which is not true as per authors’ point of view. Hence, their common nomenclature consist of Graphite Nanoplatelets (GnPs) and Graphite platelets (GPs).	[191]
17.	Graphene Oxide nanoplatelets (GONPs)	Graphene Oxide nanoplatelets (GONPs) consist of small rounded disk-shaped particles of graphene oxide in powder or solution form.	[190]
18.	Functionalized Graphene Sheets/flakes and nanoplatelets	Attaching various functional groups like epoxide, hydroxyl and carboxyl group with Graphene sheets/flakes/nanoplatelets through covalent linkage is known as Functionalized graphene sheets/flakes/nanoplatelets.	[21]
19.	Graphite nanoparticles, Graphite flakes, Graphite particles, Nano graphite platelets	Graphite flakes have the large size while graphite nanoparticles possess small size. Graphite nanoplatelets (GNPs) with a thickness in nanometer scale, can be obtained by exfoliation of natural graphite flakes.	[73]

## References

[1] Barcelo L., Kline J., Walenta G., Gartner E. (2014). Cement and carbon emissions. Mater. Struct..

[2] Kodur V., Sultan M. (2003). Effect of temperature on thermal properties of high-strength concrete. J. Mater. Civ. Eng..

[3] Javed M.F., Hafizah N., Memon S.A., Jameel M., Aslam M. (2017). Recent research on cold-formed steel beams and columns subjected to elevated temperature: A review. Constr. Build. Mater..

[4] Birchall J., Howard A., Kendall K. (1981). Flexural strength and porosity of cements. Nature.

[5] Hanehara S., Yamada K. (1999). Interaction between cement and chemical admixture from the point of cement hydration, absorption behaviour of admixture, and paste rheology. Cem. Concr. Res..

[6] Zhang M.-H., Sisomphon K., Ng T.S., Sun D.J. (2010). Effect of superplasticizers on workability retention and initial setting time of cement pastes. Constr. Build. Mater..

[7] Bessaies-Bey H., Baumann R., Schmitz M., Radler M., Roussel N. (2016). Organic admixtures and cement particles: Competitive adsorption and its macroscopic rheological consequences. Cem. Concr. Res..

[8] Flatt R.J., Houst Y.F. (2001). A simplified view on chemical effects perturbing the action of superplasticizers. Cem. Concr. Res..

[9] Rehman S.K.U., Ibrahim Z., Memon S.A., Jameel M. (2016). Nondestructive test methods for concrete bridges: A review. Constr. Build. Mater..

[10] Javed M.F., Sulong N.H.R., Memon S.A., Rehman S.K.U., Khan N.B. (2017). FE modelling of the flexural behaviour of square and rectangular steel tubes filled with normal and high strength concrete. Thin-Walled Struct..

[11] Lothenbach B., Scrivener K., Hooton R.D. (2011). Supplementary cementitious materials. Cem. Concr. Res..

[12] Richardson I., Groves G. (1997). The structure of the calcium silicate hydrate phases present in hardened pastes of white Portland cement/blast-furnace slag blends. J. Mater. Sci..

[13] Shi C., Qian J. (2000). High performance cementing materials from industrial slags—A review. Resour. Conserv. Recycl..

[14] Yoo D.-Y., Banthia N., Fujikake K., Borges P.H., Gupta R. (2017). Advanced Cementitious Materials: Mechanical Behavior, Durability, and Volume Stability. Adv. Mater. Sci. Eng..

[15] Dawood E.T., Ramli M. (2011). High strength characteristics of cement mortar reinforced with hybrid fibres. Constr. Build. Mater..

[16] Chung D.D.L. (2001). Comparison of submicron-diameter carbon filaments and conventional carbon fibers as fillers in composite materials. Carbon.

[17] Juárez C., Valdez P., Durán A., Sobolev K. (2007). The diagonal tension behavior of fiber reinforced concrete beams. Cem. Concr. Compos..

[18] Topçu İ.B., Canbaz M. (2007). Effect of different fibers on the mechanical properties of concrete containing fly ash. Constr. Build. Mater..

[19] Yoo D.-Y., Banthia N., Fujikake K., Kim Y.H., Gupta R. (2019). Fiber-Reinforced Cement Composites: Mechanical Properties and Structural Implications 2019. Adv. Mater. Sci. Eng..

[20] Konsta G.M.S., Metaxa Z.S., Shah S.P. (2010). Multi-scale mechanical and fracture characteristics and early-age strain capacity of high performance carbon nanotube/cement nanocomposites. Cem. Concr. Compos..

[21] Chuah S., Pan Z., Sanjayan J.G., Wang C.M., Duan W.H. (2014). Nano reinforced cement and concrete composites and new perspective from graphene oxide. Constr. Build. Mater..

[22] Xu Y., Fan Y. (2020). Effects of Graphene Oxide Dispersion on Salt-Freezing Resistance of Concrete. Adv. Mater. Sci. Eng..

[23] Liu C., Liu G., Ge Z., Guan Y., Cui Z., Zhou J. (2019). Mechanical and Self-Sensing Properties of Multiwalled Carbon Nanotube-Reinforced ECCs. Adv. Mater. Sci. Eng..

[24] Sanchez F., Sobolev K. (2010). Nanotechnology in concrete—A review. Constr. Build. Mater..

[25] Fraga J.L., del Campo J.M., García J.Á. Carbon nanotube-cement composites in the construction industry: 1952–2014. A state of the art review. Proceedings of the 2nd International Conference on Emerging Trends in Engineering and Technology (ICETET’2014).

[26] Han B., Sun S., Ding S., Zhang L., Yu X., Ou J. (2015). Review of nanocarbon-engineered multifunctional cementitious composites. Compos. Part A Appl. Sci. Manuf..

[27] Qureshi T.S., Panesar D.K. A review: The effect of graphene oxide on the properties of cement-based composites. Proceedings of the CSCE Annual Conference.

[28] Yang H., Cui H., Tang W., Li Z., Han N., Xing F. (2017). A critical review on research progress of graphene/cement based composites. Compos. Part A Appl. Sci. Manuf..

[29] Liu C., Huang X., Wu Y.-Y., Deng X., Liu J., Zheng Z., Hui D. (2020). Review on the research progress of cement-based and geopolymer materials modified by graphene and graphene oxide. Nanotechnol. Rev..

[30] Zhao L., Guo X., Song L., Song Y., Dai G., Liu J. (2020). An intensive review on the role of graphene oxide in cement-based materials. Constr. Build. Mater..

[31] Novoselov K., Geim A.K., Morozov S.V., Jiang D., Zhang Y., Dubonos S.V., Grigorieva I.V., Firsov A.A. (2004). Electric field effect in atomically thin carbon films. Science.

[32] Alkhateb H., Al-Ostaz A., Cheng A.H.-D., Li X. (2013). Materials genome for graphene-cement nanocomposites. J. Nanomech. Micromech..

[33] Wang B., Jiang R., Wu Z. (2016). Investigation of the mechanical properties and microstructure of graphene nanoplatelet-cement composite. Nanomaterials.

[34] Segal M. (2009). Selling graphene by the ton. Nat Nano.

[35] Novoselov K., Fal V., Colombo L., Gellert P., Schwab M., Kim K. (2012). A roadmap for graphene. Nature.

[36] Rehman S.K.U., Imtiaz L., Aslam F., Khan M.K., Haseeb M., Javed M.F., Alyousef R., Alabduljabbar H. (2020). Experimental Investigation of NaOH and KOH Mixture in SCBA-Based Geopolymer Cement Composite. Materials.

[37] Bai H., Li C., Shi G. (2011). Functional composite materials based on chemically converted graphene. Adv. Mater..

[38] Sun S., Yu X., Han B., Ou J. (2013). In situ growth of carbon nanotubes/carbon nanofibers on cement/mineral admixture particles: A review. Constr. Build. Mater..

[39] Geim A.K., Novoselov K.S. (2010). The rise of graphene. Nanoscience and Technology: A Collection of Reviews from Nature Journals.

[40] Kuilla T., Bhadra S., Yao D., Kim N.H., Bose S., Lee J.H. (2010). Recent advances in graphene based polymer composites. Prog. Polym. Sci..

[41] Kroto H.W., Heath J.R., O’Brien S.C., Curl R.F., Smalley R.E. (1985). C60: Buckminsterfullerene. Nature.

[42] Long X., Sun M.-Q., Li Z.-Q., Song X.-H. (2008). Piezo-resistive Effects in Carbon Black-filled Cement-matrix Nanocomposites. J. Wuhan Univ. Technol..

[43] Gong H., Li Z., Zhang Y., Fan R. (2009). Piezoelectric and dielectric behavior of 0-3 cement-based composites mixed with carbon black. J. Eur. Ceram. Soc..

[44] Wang P., Ding T., Xu F., Qin Y. (2004). Piezoresistivity of conductive composites filled by carbon black particles. Fuhe Cailiao Xuebao (Acta Mater. Compos. Sin.).

[45] Gao Y.-M., Shim H.-S., Hurt R.H., Suuberg E.M., Yang N.Y. (1997). Effects of carbon on air entrainment in fly ash concrete: The role of soot and carbon black. Energy Fuels.

[46] Agrawal S., Raghuveer M.S., Ramprasad R., Ramanath G. (2007). Multishell Carrier Transport in Multiwalled Carbon Nanotubes. Nanotechnol. IEEE Trans..

[47] Konsta G.M.S., Metaxa Z.S., Shah S.P. (2010). Highly dispersed carbon nanotube reinforced cement based materials. Cem. Concr. Res..

[48] Li G.Y., Wang P.M., Zhao X. (2005). Mechanical behavior and microstructure of cement composites incorporating surface-treated multi-walled carbon nanotubes. Carbon.

[49] Ma P.-C., Siddiqui N.A., Marom G., Kim J.-K. (2010). Dispersion and functionalization of carbon nanotubes for polymer-based nanocomposites: A review. Compos. Part A Appl. Sci. Manuf..

[50] Cwirzen A., Habermehl-Cwirzen K., Nasibulin A.G., Kaupinen E.I., Mudimela P.R., Penttala V. (2009). SEM/AFM studies of cementitious binder modified by MWCNT and nano-sized Fe needles. Mater. Charact..

[51] Cwirzen A. (2010). Controlling physical properties of cementitious matrixes by nanomaterials. Adv. Mater. Res..

[52] Kim J., Cote L.J., Kim F., Yuan W., Shull K.R., Huang J. (2010). Graphene Oxide Sheets at Interfaces. J. Am. Chem. Soc..

[53] Ramanathan T., Abdala A.A., Stankovich S., Dikin D.A., Herrera Alonso M., Piner R.D., Adamson D.H., Schniepp H.C., Chen X., Ruoff R.S. (2008). Functionalized graphene sheets for polymer nanocomposites. Nat. Nano.

[54] Kim H., Miura Y., Macosko C.W. (2010). Graphene/Polyurethane Nanocomposites for Improved Gas Barrier and Electrical Conductivity. Chem. Mater..

[55] Pan Z., He L., Qiu L., Korayem A.H., Li G., Zhu J.W., Collins F., Li D., Duan W.H., Wang M.C. (2015). Mechanical properties and microstructure of a graphene oxide–cement composite. Cem. Concr. Compos..

[56] Wilson N.R., Pandey P.A., Beanland R., Young R.J., Kinloch I.A., Gong L., Liu Z., Suenaga K., Rourke J.P., York S.J. (2009). Graphene oxide: Structural analysis and application as a highly transparent support for electron microscopy. ACS Nano.

[57] Zhang Y., Tan Y.-W., Stormer H.L., Kim P. (2005). Experimental observation of the quantum Hall effect and Berry’s phase in graphene. Nature.

[58] Ferrari A.C., Meyer J.C., Scardaci V., Casiraghi C., Lazzeri M., Mauri F., Piscanec S., Jiang D., Novoselov K.S., Roth S. (2006). Raman Spectrum of Graphene and Graphene Layers. Phys. Rev. Lett..

[59] Lee C., Wei X., Kysar J.W., Hone J. (2008). Measurement of the elastic properties and intrinsic strength of monolayer graphene. Science.

[60] Boehm H., Clauss A., Fischer G., Hofmann U. (1962). Surface properties of extremely thin graphite lamellae. Proceedings of Fifth Conference on Carbon.

[61] Zhu Y., Murali S., Cai W., Li X., Suk J.W., Potts J.R., Ruoff R.S. (2010). Graphene and graphene oxide: Synthesis, properties, and applications. Adv. Mater..

[62] Balandin A.A., Ghosh S., Bao W., Calizo I., Teweldebrhan D., Miao F., Lau C.N. (2008). Superior thermal conductivity of single-layer graphene. Nano Lett..

[63] Lin J., Teweldebrhan D., Ashraf K., Liu G., Jing X., Yan Z., Li R., Ozkan M., Lake R.K., Balandin A.A. (2010). Gating of Single-Layer Graphene with Single-Stranded Deoxyribonucleic Acids. Small.

[64] Geim A.K., Novoselov K.S. (2007). The rise of graphene. Nat. Mater..

[65] Lee C., Wei X., Li Q., Carpick R., Kysar J.W., Hone J. (2009). Elastic and frictional properties of graphene. Phys. Status Solidi (B).

[66] Mak K.F., Lee C., Hone J., Shan J., Heinz T.F. (2010). Atomically thin MoS 2: A new direct-gap semiconductor. Phys. Rev. Lett..

[67] Rafiee M.A., Lu W., Thomas A.V., Zandiatashbar A., Rafiee J., Tour J.M., Koratkar N.A. (2010). Graphene nanoribbon composites. ACS Nano.

[68] Grobert N. (2007). Carbon nanotubes—Becomng clean. Materialstoday.

[69] Ubertini F., Laflamme S., Ceylan H., Materazzi A.L., Cerni G., Saleem H., D’Alessandro A., Corradini A. (2014). Novel nanocomposite technologies for dynamic monitoring of structures: A comparison between cement-based embeddable and soft elastomeric surface sensors. Smart Mater. Struct..

[70] Chen J., Gao X., Xu D. (2019). Recent Advances in Characterization Techniques for the Interface in Carbon Nanotube-Reinforced Polymer Nanocomposites. Adv. Mater. Sci. Eng..

[71] Munz M., Giusca C.E., Myers-Ward R.L., Gaskill D.K., Kazakova O. (2015). Thickness-dependent hydrophobicity of epitaxial graphene. ACS Nano.

[72] Hamann H., Clemens D. (1973). Suspension Polymerization of Uniform Polymer Beads. U.S. Patent.

[73] Geng Y., Wang S.J., Kim J.-K. (2009). Preparation of graphite nanoplatelets and graphene sheets. J. Colloid Interface Sci..

[74] Li P., Yao H., Wong M., Sugiyama H., Zhang X., Sue H.-J. (2014). Thermally stable and highly conductive free-standing hybrid films based on reduced graphene oxide. J. Mater. Sci..

[75] Peyvandi A., Soroushian P., Abdol N., Balachandra A.M. (2013). Surface-modified graphite nanomaterials for improved reinforcement efficiency in cementitious paste. Carbon.

[76] Stankovich S., Piner R.D., Chen X., Wu N., Nguyen S.T., Ruoff R.S. (2006). Stable aqueous dispersions of graphitic nanoplatelets via the reduction of exfoliated graphite oxide in the presence of poly (sodium 4-styrenesulfonate). J. Mater. Chem..

[77] Lu J., Do I., Fukushima H., Lee I., Drzal L.T. (2010). Stable aqueous suspension and self-assembly of graphite nanoplatelets coated with various polyelectrolytes. J. Nanomater..

[78] Wotring E. (2014). Dispersion of Graphene Nanoplatelets in Water with Surfactant and Reinforcement of Mortar with Graphene Nanoplatelets. Ph.D. Thesis.

[79] Sixuan H. (2012). Multifunctional Graphite Nanoplatelets (GNP) Reinforced Cementitious Composites. Master’s Thesis.

[80] Sharif M.S., Fard F.G., Khatibi E., Sarpoolaky H. (2009). Dispersion and stability of carbon black nanoparticles, studied by ultraviolet–visible spectroscopy. J. Taiwan Inst. Chem. Eng..

[81] Silva R.A., de Castro Guetti P., da Luz M.S., Rouxinol F., Gelamo R.V. (2017). Enhanced properties of cement mortars with multilayer graphene nanoparticles. Constr. Build. Mater..

[82] Han B., Zheng Q., Sun S., Dong S., Zhang L., Yu X., Ou J. (2017). Enhancing mechanisms of multi-layer graphenes to cementitious composites. Compos. Part A Appl. Sci. Manuf..

[83] Li J., Sham M.L., Kim J.-K., Marom G. (2007). Morphology and properties of UV/ozone treated graphite nanoplatelet/epoxy nanocomposites. Compos. Sci. Technol..

[84] Mehrali M., Sadeghinezhad E., Latibari S.T., Kazi S.N., Mehrali M., Zubir M.N.B.M., Metselaar H.S.C. (2014). Investigation of thermal conductivity and rheological properties of nanofluids containing graphene nanoplatelets. Nanoscale Res. Lett..

[85] Jiang L., Gao L., Sun J. (2003). Production of aqueous colloidal dispersions of carbon nanotubes. J. Colloid Interface Sci..

[86] Liu M., Horrocks A. (2002). Effect of carbon black on UV stability of LLDPE films under artificial weathering conditions. Polym. Degrad. Stab..

[87] Jäger C., Henning T., Schlögl R., Spillecke O. (1999). Spectral properties of carbon black. J. Non-Cryst. Solids.

[88] Aunkor M., Mahbubul I., Saidur R., Metselaar H. (2015). Deoxygenation of graphene oxide using household baking soda as a reducing agent: A green approach. RSC Adv..

[89] Lei Y., Tang Z., Liao R., Guo B. (2011). Hydrolysable tannin as environmentally friendly reducer and stabilizer for graphene oxide. Green Chem..

[90] Li J., Xiao G., Chen C., Li R., Yan D. (2013). Superior dispersions of reduced graphene oxide synthesized by using gallic acid as a reductant and stabilizer. J. Mater. Chem. A.

[91] Mei X., Ouyang J. (2011). Ultrasonication-assisted ultrafast reduction of graphene oxide by zinc powder at room temperature. Carbon.

[92] Xu L.Q., Yang W.J., Neoh K.-G., Kang E.-T., Fu G.D. (2010). Dopamine-induced reduction and functionalization of graphene oxide nanosheets. Macromolecules.

[93] Wang Y., Shi Z., Yin J. (2011). Facile synthesis of soluble graphene via a green reduction of graphene oxide in tea solution and its biocomposites. ACS Appl. Mater. Interfaces.

[94] Thakur S., Karak N. (2012). Green reduction of graphene oxide by aqueous phytoextracts. Carbon.

[95] Chen D., Li L., Guo L. (2011). An environment-friendly preparation of reduced graphene oxide nanosheets via amino acid. Nanotechnology.

[96] Khai T.V., Kwak D.S., Kwon Y.J., Cho H.Y., Huan T.N., Chung H., Ham H., Lee C., Dan N.V., Tung N.T. (2013). Direct production of highly conductive graphene with a low oxygen content by a microwave-assisted solvothermal method. Chem. Eng. J..

[97] Peng H., Meng L., Niu L., Lu Q. (2012). Simultaneous reduction and surface functionalization of graphene oxide by natural cellulose with the assistance of the ionic liquid. J. Phys. Chem. C.

[98] Tran D.N., Kabiri S., Losic D. (2014). A green approach for the reduction of graphene oxide nanosheets using non-aromatic amino acids. Carbon.

[99] Bose S., Kuila T., Mishra A.K., Kim N.H., Lee J.H. (2012). Dual role of glycine as a chemical functionalizer and a reducing agent in the preparation of graphene: An environmentally friendly method. J. Mater. Chem..

[100] Pham V.H., Pham H.D., Dang T.T., Hur S.H., Kim E.J., Kong B.S., Kim S., Chung J.S. (2012). Chemical reduction of an aqueous suspension of graphene oxide by nascent hydrogen. J. Mater. Chem..

[101] Gurunathan S., Han J., Kim J.H. (2013). Humanin: A novel functional molecule for the green synthesis of graphene. Colloids Surf. B Biointerfaces.

[102] Zhang J., Yang H., Shen G., Cheng P., Zhang J., Guo S. (2010). Reduction of graphene oxide via L-ascorbic acid. Chem. Commun..

[103] Khanra P., Kuila T., Kim N.H., Bae S.H., Yu D.-S., Lee J.H. (2012). Simultaneous bio-functionalization and reduction of graphene oxide by baker’s yeast. Chem. Eng. J..

[104] Zhu C., Guo S., Fang Y., Dong S. (2010). Reducing sugar: New functional molecules for the green synthesis of graphene nanosheets. ACS Nano.

[105] Liu S., Tian J., Wang L., Sun X. (2011). A method for the production of reduced graphene oxide using benzylamine as a reducing and stabilizing agent and its subsequent decoration with Ag nanoparticles for enzymeless hydrogen peroxide detection. Carbon.

[106] Leng Y. (2009). Materials Characterization: Introduction to Microscopic and Spectroscopic Methods.

[107] Sharma S., Kothiyal N. (2016). Comparative effects of pristine and ball-milled graphene oxide on physico-chemical characteristics of cement mortar nanocomposites. Constr. Build. Mater..

[108] Mollah M.Y.A., Yu W., Schennach R., Cocke D.L. (2000). A Fourier transform infrared spectroscopic investigation of the early hydration of Portland cement and the influence of sodium lignosulfonate. Cem. Concr. Res..

[109] Ortego J.D., Jackson S., Yu G.S., McWhinney H., Cocke D.L. (1989). Solidification of hazardous substances-a TGA and FTIR study of Portland cement containing metal nitrates. J. Environ. Sci. Health Part A.

[110] Horgnies M., Chen J., Bouillon C. (2013). Overview about the use of Fourier Transform Infrared spectroscopy to study cementitious materials. Proceedings of the 6th International Conference on Computational Methods and Experiments in Materials Characterization.

[111] Bensted J., Varma S.P. (1974). Some applications of infrared and Raman spectroscopy in cement chemistry. Part 3-hydration of Portland cement and its constituents. Cem. Technol..

[112] Naeimi H., Golestanzadeh M. (2015). Microwave-assisted synthesis of 6,6′-(aryl (alkyl) methylene) bis (2,4-dialkylphenol) antioxidants catalyzed by multi-sulfonated reduced graphene oxide nanosheets in water. New J. Chem..

[113] Smith B.C. (2011). Fundamentals of Fourier Transform Infrared Spectroscopy.

[114] Murugan M., Santhanam M., Gupta S.S., Pradeep T., Shah S.P. (2016). Influence of 2D rGO nanosheets on the properties of OPC paste. Cem. Concr. Compos..

[115] Lv S., Ma Y., Qiu C., Sun T., Liu J., Zhou Q. (2013). Effect of graphene oxide nanosheets of microstructure and mechanical properties of cement composites. Constr. Build. Mater..

[116] Cui H., Yan X., Tang L., Xing F. (2017). Possible pitfall in sample preparation for SEM analysis—A discussion of the paper “Fabrication of polycarboxylate/graphene oxide nanosheet composites by copolymerization for reinforcing and toughening cement composites” by Lv et al. Cem. Concr. Compos..

[117] Cao M.-L., Zhang H.-X., Zhang C. (2016). Effect of graphene on mechanical properties of cement mortars. J. Cent. South Univ..

[118] Rehman S.K.U., Ibrahim Z., Memon S.A., Javed M.F., Khushnood R.A. (2017). A sustainable graphene based cement composite. Sustainability.

[119] Zhang Y., Kong X., Gao L., Lu Z., Zhou S., Dong B., Xing F. (2016). In-situ measurement of viscoelastic properties of fresh cement paste by a microrheology analyzer. Cem. Concr. Res..

[120] Nazar S., Yang J., Thomas B.S., Azim I., Ur Rehman S.K. (2020). Rheological properties of cementitious composites with and without nano-materials: A comprehensive review. J. Clean. Prod..

[121] Ferraris C.F. Measurement of the rheological properties of cement paste: A new approach. Proceedings of the International RILEM Conference the Role of Admixtures in High Performance Concrete.

[122] Kawashima S., Hou P., Corr D.J., Shah S.P. (2013). Modification of cement-based materials with nanoparticles. Cem. Concr. Compos..

[123] Ormsby R., McNally T., Mitchell C., Halley P., Martin D., Nicholson T., Dunne N. (2011). Effect of MWCNT addition on the thermal and rheological properties of polymethyl methacrylate bone cement. Carbon.

[124] Bilal H., Yaqub M., Rehman S.K.U., Abid M., Alyousef R., Alabduljabbar H., Aslam F. (2019). Performance of Foundry Sand Concrete under Ambient and Elevated Temperatures. Materials.

[125] Shang Y., Zhang D., Yang C., Liu Y., Liu Y. (2015). Effect of graphene oxide on the rheological properties of cement pastes. Constr. Build. Mater..

[126] Wang Q., Wang J., Lv C.-X., Cui X.-Y., Li S.-Y., Wang X. (2016). Rheological behavior of fresh cement pastes with a graphene oxide additive. New Carbon Mater..

[127] Wang Q., Cui X., Wang J., Li S., Lv C., Dong Y. (2017). Effect of fly ash on rheological properties of graphene oxide cement paste. Constr. Build. Mater..

[128] Rehman S.K.U., Ibrahim Z., Memon S.A., Aunkor M., Hossain T., Javed M.F., Mehmood K., Shah S.M.A. (2018). Influence of Graphene Nanosheets on Rheology, Microstructure, Strength Development and Self-Sensing Properties of Cement Based Composites. Sustainability.

[129] Yahia A., Khayat K.H. (2001). Analytical models for estimating yield stress of high-performance pseudoplastic grout. Cem. Concr. Res..

[130] Nehdi M., Rahman M.A. (2004). Estimating rheological properties of cement pastes using various rheological models for different test geometry, gap and surface friction. Cem. Concr. Res..

[131] Rehman S.K.U., Ibrahim Z., Jameel M., Memon S.A., Javed M.F., Aslam M., Mehmood K., Nazar S. (2018). Assessment of Rheological and Piezoresistive Properties of Graphene based Cement Composites. Int. J. Concr. Struct. Mater..

[132] Wengui L., Li X., Chen S.J., Liu Y.M., Duan W.H., Shah S.P. (2017). Effects of graphene oxide on early-age hydration and electrical resistivity of Portland cement paste. Constr. Build. Mater..

[133] Shenghua L., Ma Y., Qiu C., Zhou Q. (2013). Regulation of GO on cement hydration crystals and its toughening effect. Mag. Concr. Res..

[134] Mokhtar M., Abo-El-Enein S., Hassaan M., Morsy M., Khalil M. (2017). Mechanical performance, pore structure and micro-structural characteristics of graphene oxide nano platelets reinforced cement. Constr. Build. Mater..

[135] Wang M., Wang R., Yao H., Farhan S., Zheng S., Du C. (2016). Study on the three dimensional mechanism of graphene oxide nanosheets modified cement. Constr. Build. Mater..

[136] Kothiyal N., Sharma S., Mahajan S., Sethi S. (2016). Characterization of reactive graphene oxide synthesized from ball–milled graphite: Its enhanced reinforcing effects on cement nanocomposites. J. Adhes. Sci. Technol..

[137] Shamsaei E., de Souza F.B., Yao X., Benhelal E., Akbari A., Duan W. (2018). Graphene-based nanosheets for stronger and more durable concrete: A review. Constr. Build. Mater..

[138] Sharma S., Kothiyal N. (2015). Influence of graphene oxide as dispersed phase in cement mortar matrix in defining the crystal patterns of cement hydrates and its effect on mechanical, microstructural and crystallization properties. RSC Adv..

[139] Abrishami M.E., Zahabi V. (2016). Reinforcing graphene oxide/cement composite with NH2 functionalizing group. Bull. Mater. Sci..

[140] Lv S., Deng L., Yang W., Zhou Q., Cui Y. (2016). Fabrication of polycarboxylate/graphene oxide nanosheet composites by copolymerization for reinforcing and toughening cement composites. Cem. Concr. Compos..

[141] Farooq F., Rahman S.K.U., Akbar A., Khushnood R.A., Javed M.F., alyousef R., alabduljabbar H., aslam F. (2020). A comparative study on performance evaluation of hybrid GNPs/CNTs in conventional and self-compacting mortar. Alex. Eng. J..

[142] Farooq F., Akbar A., Khushnood R.A., Muhammad W.L., Rehman S.K., Javed M.F. (2020). Experimental Investigation of Hybrid Carbon Nanotubes and Graphite Nanoplatelets on Rheology, Shrinkage, Mechanical, and Microstructure of SCCM. Materials.

[143] Hou D., Lu Z., Li X., Ma H., Li Z. (2017). Reactive molecular dynamics and experimental study of graphene-cement composites: Structure, dynamics and reinforcement mechanisms. Carbon.

[144] Lv S., Liu J., Sun T., Ma Y., Zhou Q. (2014). Effect of GO nanosheets on shapes of cement hydration crystals and their formation process. Constr. Build. Mater..

[145] Lv S., Ting S., Liu J., Zhou Q. (2014). Use of graphene oxide nanosheets to regulate the microstructure of hardened cement paste to increase its strength and toughness. CrystEngComm.

[146] Wang Q., Wang J., Lu C.-X., Liu B.-W., Zhang K., Li C.-Z. (2015). Influence of graphene oxide additions on the microstructure and mechanical strength of cement. New Carbon Mater..

[147] Metaxa Z.S. (2016). Exfoliated graphene nanoplatelet cement-based nanocomposites as piezoresistive sensors: Influence of nanoreinforcement lateral size on monitoring capability. Cienc. Tecnol. Dos Mater..

[148] Zhao L., Guo X., Ge C., Li Q., Guo L., Shu X., Liu J. (2016). Investigation of the effectiveness of PC@ GO on the reinforcement for cement composites. Constr. Build. Mater..

[149] Lu Z., Hou D., Meng L., Sun G., Lu C., Li Z. (2015). Mechanism of cement paste reinforced by graphene oxide/carbon nanotubes composites with enhanced mechanical properties. RSC Adv..

[150] Sun X., Wu Q., Zhang J., Qing Y., Wu Y., Lee S. (2017). Rheology, curing temperature and mechanical performance of oil well cement: Combined effect of cellulose nanofibers and graphene nano-platelets. Mater. Des..

[151] Zhou C., Li F., Hu J., Ren M., Wei J., Yu Q. (2017). Enhanced mechanical properties of cement paste by hybrid graphene oxide/carbon nanotubes. Constr. Build. Mater..

[152] Gong K., Pan Z., Korayem A.H., Qiu L., Li D., Collins F., Wang C.M., Duan W.H. (2014). Reinforcing effects of graphene oxide on portland cement paste. J. Mater. Civ. Eng..

[153] Zhao L., Guo X., Ge C., Li Q., Guo L., Shu X., Liu J. (2017). Mechanical behavior and toughening mechanism of polycarboxylate superplasticizer modified graphene oxide reinforced cement composites. Compos. Part B Eng..

[154] Qian Y., Abdallah M.Y., Kawashima S. (2015). Characterization of Cement-Based Materials Modified with Graphene-Oxide. Nanotechnology in Construction.

[155] Sun H., Ling L., Ren Z., Memon S.A., Xing F. (2020). Effect of graphene oxide/graphene hybrid on mechanical properties of cement mortar and mechanism investigation. Nanomaterials.

[156] Zhao L., Guo X., Liu Y., Ge C., Guo L., Shu X., Liu J. (2017). Synergistic effects of silica nanoparticles/polycarboxylate superplasticizer modified graphene oxide on mechanical behavior and hydration process of cement composites. RSC Adv..

[157] Mohammed A., Sanjayan J., Duan W., Nazari A. (2016). Graphene Oxide Impact on Hardened Cement Expressed in Enhanced Freeze–Thaw Resistance. J. Mater. Civ. Eng..

[158] Kaur R., Kothiyal N.C., Arora H. (2020). Studies on combined effect of superplasticizer modified graphene oxide and carbon nanotubes on the physico-mechanical strength and electrical resistivity of fly ash blended cement mortar. J. Build. Eng..

[159] Tong T., Fan Z., Liu Q., Wang S., Tan S., Yu Q. (2016). Investigation of the effects of graphene and graphene oxide nanoplatelets on the micro-and macro-properties of cementitious materials. Constr. Build. Mater..

[160] Rhee I., Lee J.S., Kim Y.A., Kim J.H., Kim J.H. (2016). Electrically conductive cement mortar: Incorporating rice husk-derived high-surface-area graphene. Constr. Build. Mater..

[161] Rhee I., Kim Y.A., Shin G.-O., Kim J.H., Muramatsu H. (2015). Compressive strength sensitivity of cement mortar using rice husk-derived graphene with a high specific surface area. Constr. Build. Mater..

[162] Devasena M., Karthikeyan J. (2015). Investigation on strength properties of graphene oxide concrete. Int. J. Eng. Sci. Invent. Res. Dev..

[163] Duarte F., Ferreira A. (2016). Energy harvesting on road pavements: State of the art. Proc. Inst. Civ. Eng.-Energy.

[164] Ghosh S., Harish S., Rocky K.A., Ohtaki M., Saha B.B. (2019). Graphene enhanced thermoelectric properties of cement based composites for building energy harvesting. Energy Build..

[165] Balandin A.A. (2011). Thermal properties of graphene and nanostructured carbon materials. Nat. Mater..

[166] Wei J., Zhao L., Zhang Q., Nie Z., Hao L. (2018). Enhanced thermoelectric properties of cement-based composites with expanded graphite for climate adaptation and large-scale energy harvesting. Energy Build..

[167] Wei J., Fan Y., Zhao L., Xue F., Hao L., Zhang Q. (2018). Thermoelectric properties of carbon nanotube reinforced cement-based composites fabricated by compression shear. Ceram. Int..

[168] Tzounis L., Liebscher M., Fuge R., Leonhardt A., Mechtcherine V. (2019). P- and n-type thermoelectric cement composites with CVD grown p- and n-doped carbon nanotubes: Demonstration of a structural thermoelectric generator. Energy Build..

[169] Cooper D.R., D’Anjou B., Ghattamaneni N., Harack B., Hilke M., Horth A., Majlis N., Massicotte M., Vandsburger L., Whiteway E. (2012). Experimental review of graphene. ISRN Condens. Matter Phys..

[170] Zhao H., Bai J. (2015). Highly sensitive piezo-resistive graphite nanoplatelet–carbon nanotube hybrids/polydimethylsilicone composites with improved conductive network construction. ACS Appl. Mater. Interfaces.

[171] Han B.G., Han B.Z., Ou J.P. (2009). Experimental study on use of nickel powder-filled Portland cement-based composite for fabrication of piezoresistive sensors with high sensitivity. Sens. Actuators A Phys..

[172] Han B., Guan X., Ou J. (2007). Electrode design, measuring method and data acquisition system of carbon fiber cement paste piezoresistive sensors. Sens. Actuators A Phys..

[173] Li G.Y., Wang P.M., Zhao X. (2007). Pressure-sensitive properties and microstructure of carbon nanotube reinforced cement composites. Cem. Concr. Compos..

[174] Le J.-L., Du H., Dai Pang S. (2014). Use of 2D Graphene Nanoplatelets (GNP) in cement composites for structural health evaluation. Compos. Part B Eng..

[175] Valdes L.B. (1954). Resistivity measurements on germanium for transistors. Proc. IRE.

[176] Chung D.D.L. (2002). Piezoresistive cement-based materials for strain sensing. J. Intell. Mater. Syst. Struct..

[177] Qu J., Han B. (2008). Piezoresistive cement-based strain sensors and self-sensing concrete components. J. Intell. Mater. Syst. Struct..

[178] Li H., Xiao H.-G., Ou J.-P. (2004). A study on mechanical and pressure-sensitive properties of cement mortar with nanophase materials. Cem. Concr. Res..

[179] Yu X., Kwon E. (2009). A carbon nanotube/cement composite with piezoresistive properties. Smart Mater. Struct..

[180] Du H., Quek S.T., Dai Pang S. Smart multifunctional cement mortar containing graphite nanoplatelet. Proceedings of the SPIE Smart Structures and Materials+ Nondestructive Evaluation and Health Monitoring.

[181] Lahiri I., Verma V.P., Choi W. (2011). An all-graphene based transparent and flexible field emission device. Carbon.

[182] Endo M., Hayashi T., Ahm Kim Y., Terrones M., Dresselhaus M.S. (2004). Applications of carbon nanotubes in the twenty–first century. Philos. Trans. R. Soc. Lond. Ser. A Math. Phys. Eng. Sci..

[183] Horszczaruk E., Mijowska E., Kalenczuk R.J., Aleksandrzak M., Mijowska S. (2015). Nanocomposite of cement/graphene oxide–Impact on hydration kinetics and Young’s modulus. Constr. Build. Mater..

[184] Raidongia K., Tan A.T., Huang J. (2014). Graphene oxide: Some new insights into an old material. Carbon Nanotubes and Graphene.

[185] Iris K., Xiong X., Tsang D.C., Ng Y.H., Clark J.H., Fan J., Zhang S., Hu C., Ok Y.S. (2019). Graphite oxide-and graphene oxide-supported catalysts for microwave-assisted glucose isomerisation in water. Green Chem..

[186] Stankovich S., Dikin D.A., Piner R.D., Kohlhaas K.A., Kleinhammes A., Jia Y., Wu Y., Nguyen S.T., Ruoff R.S. (2007). Synthesis of graphene-based nanosheets via chemical reduction of exfoliated graphite oxide. Carbon.

[187] Chandrasekaran S., Sato N., Tölle F., Mülhaupt R., Fiedler B., Schulte K. (2014). Fracture toughness and failure mechanism of graphene based epoxy composites. Compos. Sci. Technol..

[188] Harris P.J. (2005). New perspectives on the structure of graphitic carbons. Crit. Rev. Solid State Mater. Sci..

[189] Huang X., Yin Z., Wu S., Qi X., He Q., Zhang Q., Yan Q., Boey F., Zhang H. (2011). Graphene-based materials: Synthesis, characterization, properties, and applications. Small.

[190] Roni Peleg. https://www.ossila.com/pages/introduction-to-graphene.

[191] Cataldi P., Athanassiou A., Bayer I.S. (2018). Graphene nanoplatelets-based advanced materials and recent progress in sustainable applications. Appl. Sci..

